# Gamma Irradiation and the Radiation Shielding Characteristics: For the Lead Oxide Doped the Crosslinked Polystyrene-b-Polyethyleneglycol Block Copolymers and the Polystyrene-b-Polyethyleneglycol-Boron Nitride Nanocomposites

**DOI:** 10.3390/polym13193246

**Published:** 2021-09-24

**Authors:** Zehra Merve Cinan, Burcu Erol, Taylan Baskan, Saliha Mutlu, Sevil Savaskan Yilmaz, Ahmet Hakan Yilmaz

**Affiliations:** 1 Faculty of Sciences, Department of Physics, Karadeniz Technical University, Trabzon 61080, Turkey; m_cinan@ktu.edu.tr (Z.M.C.); taylanbaskan@ktu.edu.tr (T.B.); 2 Faculty of Arts and Sciences, Department of Physics, Recep Tayyip Erdoğan University, Rize 53100, Turkey; burcu.karayunus@gmail.com; 3 Faculty of Sciences, Department of Chemistry, Karadeniz Technical University, Trabzon 61080, Turkey; salihamutlu78@gmail.com (S.M.); sevilsyilmaz@gmail.com (S.S.Y.)

**Keywords:** attenuation coefficients, block copolymer, boron nitride, gamma irradiation, lead oxide, nanocomposite, polystyrene-b-polyethyleneglycol, radiation protection, shielding material

## Abstract

This work aimed to research the efficiency of gamma irradiation and shielding characteristics on the lead oxide (PbO) doped the crosslinked polystyrene-b-polyethyleneglycol (PS-b-PEG) block copolymers and polystyrene-b-polyethyleneglycol-boron nitride (PS-b-PEG-BN) nanocomposites materials. The crosslinked PS-b-PEG block copolymers and PS-b-PEG-BN nanocomposites mixed with different percentage rates of PbO were used to research gamma-ray shielding characteristics. The synthesis of the copolymer was done by emulsion polymerization methods. The characterization and morphological analyses of irradiated samples were explored handling with the Nuclear Magnetic Resonance (NMR), Fourier Transform Infrared Spectroscopy (FTIR), Gel Permeation Chromatography (GPC), Thermogravimetric Analysis (TGA), and Scanning Electron Microscope (SEM) methods. The gamma-rays that were emitted from the E 152u source were observed with a High Purity Germanium (HPGe) detector system and examined with a GammaVision computer program. Our samples, including the different percentage rates of the PS-b-PEG (1000, 1500, 10,000), BN, and PbO, were irradiated in various gamma-ray photon energy regions (from 121.78 keV to 1408.01 keV). Then, Linear-Mass Attenuation Coefficients (LACs-MACs), Half-Tenth Value Layer (HVL), Mean Free Path (MFP), and Radiation Protection Efficiency (RPE) values of the samples were calculated. Via crosschecking the acquired data from samples with and without PbO and BN, it was observed that, if the different percentage rates by weight nano-powder of PbO and BN are added in the polymer mixture, it can be used as a convenient shielding material against gamma rays.

## 1. Introduction

Today, the use of radiation sources has increased in parallel with technological development. In addition to defining the various radiation characteristics and understanding their potential benefits, the potential harms that may occur if affected by radiation are also revealed. Considering the increase in the number of nuclear power plants, the possibilities of using nuclear weapons in wars, the radiation-emitting device, and the increase in the radioisotopes used, it has emerged that more effective measures should be taken to protect from radiation. For this purpose, studies on radiation shielding materials and designs for protection from radiation have also increased [1,2,3,4].

Gamma rays, also popularly known as gamma radiation, have a short wavelength which makes the gamma rays good energy and the most powerful among other electromagnetic waves. These strong electromagnetic waves are naturally occurring, and they traverse the universe at great distances. Gamma rays are used in treating serious health problems such as cancer. Radiation oncology or radiation therapy makes use of gamma rays to control or kill malignant tumor cells in patients. However, the use of these powerful forms of energy does not focus mainly on destroying cancer cells, as healthy cells are also killed through the process. When healthy cells are also destroyed, there may be a possibility for side effects to happen. One of the most horrific dangers of gamma rays comes when these strong forms of energy are utilized in large doses. Gamma rays are also the energy that is involved in some of the most tragic accidents, such as the accident that occurred in Fukushima and the horrific earthquake that happened in 2011. On these considerations, studies aimed at developing efficient radiation shielding materials are of great interest and could have a great impact and attract the interest of most of the scientific community.

Since it is not possible to give up radiation, protection measures should be taken to reduce its harmful effects. The basic idea in radiation protection is to be exposed to radiation as low as possible. The As Low As Reasonably Achievable (ALARA) principle expresses the necessity of minimizing the dose emitted and exposed from any radioactive source [5,6,7,8]. It covers a regulation that is not only for safety purposes but also includes the necessary legislative procedures for all radiation protection programs. According to this principle, there are three basic elements in radiation protection: as short time as possible, as long as possible distance, and shielding. The most effective factor in reducing the radiation dose and its harmful effects is to put the barrier between the radiation sources. The material that creates this barrier is called shielding material [9,10,11,12]. The material to be used in the shielding design differs according to the type and energy of the radiation to be shielded. While alpha particles can be blocked with paper, it is sufficient to shield beta particles, which are more penetrating, with a thin aluminum layer. The penetration of gamma rays is 100 times higher than beta rays and 10,000 times more than alpha particles, depending on the energy of the radiating source. Stopping electromagnetic radiation, which has such a high penetration, can only be achieved with the use of special materials [13,14,15,16]. The radiation propagated by some sources is so powerful that you can be subjected to the effect even though you cannot observe it from long distances. It can be maintained from vigorous radioactive influences only by strong shielding to absorb radiation [17,18,19].

In parallel with technological developments, materials for different purposes have been increasingly needed in various sectors. Many properties can be requested at the same time in a material, but there may not be a material that shows all the desired properties by itself. A high-performance material with properties such as strength, abrasion resistance, impact resistance, and lightness may be required. All of these properties are difficult to achieve with a single type of material such as metal, ceramic, or polymer. In this case, materials with good properties, each of which is different, are brought together to form a phase of the composite and composite materials are developed. In general, the physical and chemical properties of the composite material are macroscopically different, formed by combining the best properties of two or more materials that do not mix, or by combining them with appropriate methods at the macro level, to reveal a new feature that does not exist alone in any of the components these are materials with superior properties. In other words, they can also be called materials consisting of different types of materials or phases that are brought together to obtain superior properties by correcting each other’s weak aspects. Accordingly, the material exhibits a heterogeneous material microscopically but behaves like a macroscopically homogeneous material.

Concerns about the amount of radiation that we can be exposed to due to various conditions and the expansion of research and application areas of nuclear physics day by day, the significance in radiation shielding has increased. In other words, we are in an age where we have to dabble with plenty of issues of radioactive substances and instruments in many research areas as a global development plan in which we turn to alternative and environmentally friendly energy productions.

Polymers are large molecules formed by the joining together of a lot of monomers. A polymer molecule can contain tens, hundreds, or even thousands of monomer units. The most important features of polymer composites are that they are easy to form, have low density compared to metals, have superior surface quality, and increase corrosion resistance. In addition to many advantages such as high strength, size and thermal stability, hardness, and abrasion resistance, polymer composites can compete with metals in terms of durability and hardness. In addition to all these features, their lightness also gives polymer composite materials superiority in many areas. For these reasons, polymer composite-based materials have been the focus point of scientific interest and active multidisciplinary and industrial research groups because of their interesting properties and potential [20,21,22]. In addition, many explorers have remarkably conducted polymer composite-based radiation-absorbing substances [23,24,25,26,27]. The fact that polymers can be with ease manufactured in a coating style, characterized with an extruder, or shaped for textural parts is substantial supremacy for gamma-irradiation shielding enforcements of polymer-based compounds. Polymer-based structures include major quantities of hydrogen atoms. Because hydrogen atoms have a low mass, they are very effective at decelerate high-energy particles such as neutrons.

Lead, which ranks 34th among the elements found on earth, has an atomic number of 82, and an atomic weight of 207.21 g/mol. Today, lead and lead-doped materials are widely used for shielding to protect nuclear and radiation workers from radiation, due to their high radiation retention and low cost. A lot of substances used for gamma-irradiation shieldings contain elements such as lead, titanium, bismuth, concrete, cement, paraffin, and polymers. PbO was selected as a contribution material to perform better radiation absorber and shielding characteristics since gamma rays are one of the best absorbers by intense substances. The application of metal oxide is owing to the aspiration to cultivate the absorbing qualities for elevated-energy gamma irradiations. Metal oxide additives enrich research by facilitating the capture of gamma rays of the prepared materials and improving their radiation shielding properties.

A lot of research has been put forward in which the structure of shielding substances is enriched by the addition of PbO [28,29,30,31,32,33,34,35,36,37,38]. Furthermore, in recent years, many studies have been carried out about boron nitride-doped radiation shielding materials to protect from neutron radiations. Developed materials should show more than radiation shielding functions, as they are an important material candidate for space studies. In other words, multifunctional boron nitride-doped materials with high hydrogen absorption properties are an important research area because they show effective shielding properties [39,40,41,42].

In this study, we have used the crosslinked PS-b-PEG block copolymers as a polymeric matrix along with BN and PbO as the radiation absorbing functional substance and to reduce high-energy radiation gamma rays. The LACs and MACs of the crosslinked PS-b-PEG block copolymers-BN-PbO nanocomposites were researched for various items in the linked photon energy region and compilation of the gamma-ray attenuation coefficients. We observed admissible consistency between the experimental and theoretical LACs and MACs of our samples, and the measured and calculated values show changes with the change of the polymer type used to improve the gamma radiation shielding materials. For the same purpose, the HVL, TVL, MFP, and RPE values of the crosslinked PS-b-PEG block copolymers-BN-PbO nanocomposites were investigated in the relevant photon energy region for the gamma-ray attenuation characteristics [43,44,45,46,47,48,49,50,51]. These results are a very important marker of the radiation shielding characteristic of the material concerned.

The composite material will sustain the safeness of both clinical personnel and patients in the radiation zone to lessen the needless radiation sustain of tissues and organs throughout the cure. The crosslinked PS-b-PEG block copolymers, BN, and PbO materials were developed for different applications, such as gamma radiation protective substances with the elastomeric and pliable structures for the conveyance of radioactive sources, insulation substances for the radioactive waste management facilities, and the building industry.

Our nanocomposite substances will maintain the secure application in the radiation area by both radiation employees and patients throughout radiation implementations in medical and nuclear reactors to diminish redundant exposure of tissues and organs. This inquisitorial is dedicated to the investigation of the expectations for utilization of new polymer and metal oxide contributions for enhancement of its radiation durabilities and shielding qualities. In addition, as we mentioned before, polymers have been used as an important base substance for radiation shieldings because they contain high hydrogen atoms.

## 2. Materials and Methods

The synthesis and characterization of the polymers in all materials included in this research were developed by our team at Physical Chemistry Laboratory, Department of Chemistry, Karadeniz Technical University.

### 2.1. Materials

Styrene (C_8_H_8_) was an Aldrich product (Sigma-Aldrich Inc., St. Louis, MO, USA) and was washed 3 times with 10% aqueous sodium hydroxide (NaOH) solution, dried over calcium dichloride (CaCl_2_), and distilled under reduced pressure over calcium hydride (CaH_2_) before use. Hydroquinone (C_6_H_6_O_2_), dichloroethane (C_2_H_4_Cl_2_), methacryloyl chloride (C_4_H_5_ClO), chloroform (CHCl_3_), sodium hydrogen carbonate (NaHCO_3_), and lead oxide (PbO) were Merck products (Merck & Co. Inc., Kenilworth, NJ, USA). Boron nitride (BN) powder was 1 μm in size and Aldrich product. PEG-1000, PEG-1500, PEG-10,000 were Merck products. The PEG DM-1000, PEG DM-1500, PEG DM-10,000 macro crosslinkers were synthesized from PEG-1000, PEG-1500, PEG-10,000 polymers by our group. PEG-DM is the abbreviation of poly (ethylene glycol dimethylmethacrylate) in PEG DM-1000, PEG DM-1500, PEG DM-10,000 [52,53].

### 2.2. Synthesis

#### 2.2.1. Synthesis of the PEG Macro Crosslinker

**Scheme 1:**
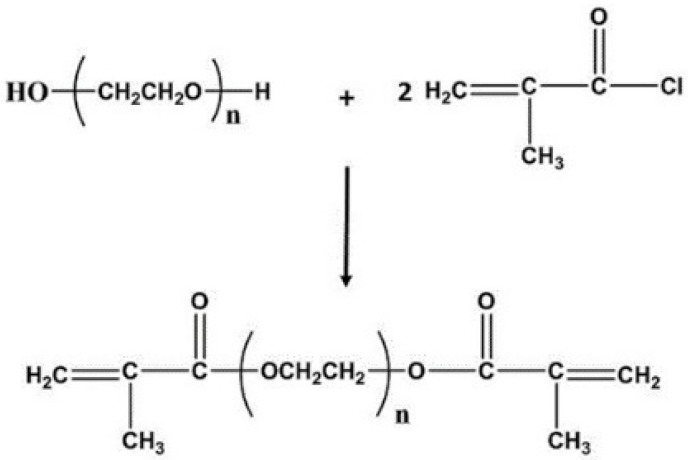
The vinylization reaction scheme of polyethyleneglycol with methacryloyl chloride.

**Figure 1:**
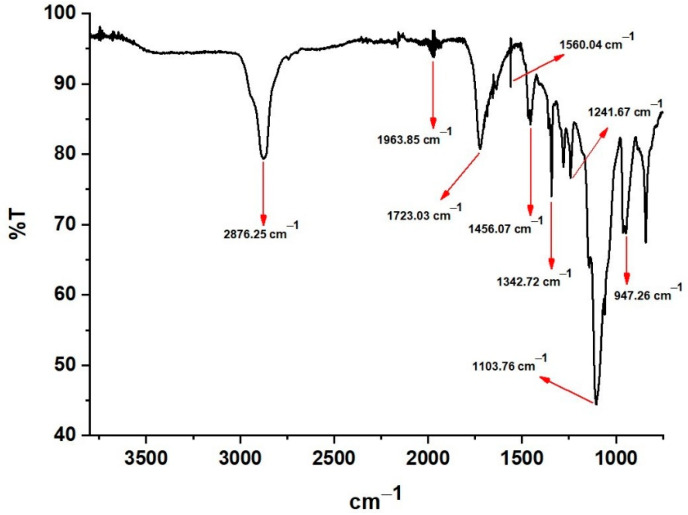
The FTIR spectrum of PEG-DM Macro Crosslinker.

**Figure 2:**
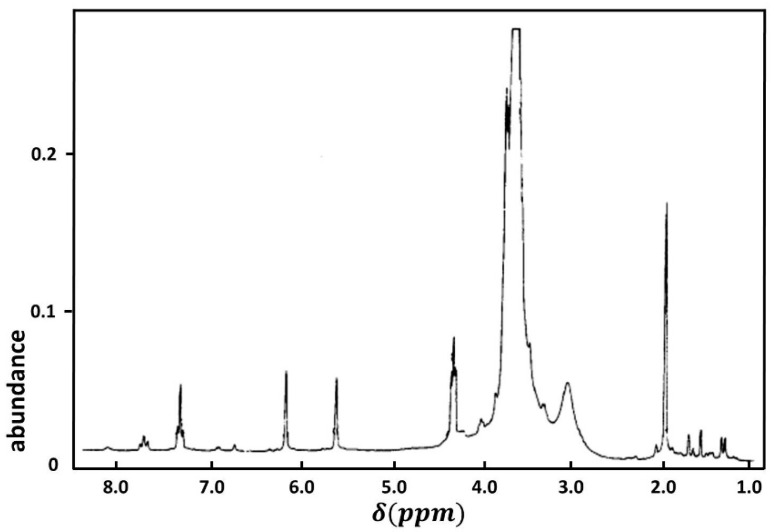
^1^H NMR spectrum of PEG-DM Macro Crosslinker (in CDCl_3_).

0.04 mol of PEG-1000 or PEG-1500 and PEG-10,000 polymers were placed in a 300 mL two-neck round bottom flask and dissolved by adding 50 mL of C_2_H_2_Cl_2_. 1–2 mg of hydroquinone and 8.5 mL of pyridine were added to the flask. 8.5 mL of methacryloyl chloride dissolved in 15–20 mL of C_2_H_4_Cl_2_ was added to the balloon slowly by mixing and cooling. As the reaction progressed, clouding and warming of the balloon were observed. It was filtered after being kept in the refrigerator overnight. The solvent was evaporated from the rotary evaporator. After adding 300 mL of CHCl_3_ to the solution, it was washed with saturated NaHCO_3_ solution by gently shaking. It was dried over anhydrous Na_2_SO_4_ [52,53]. The vinylation reaction mechanism is given in Scheme 1. Figure 1 and Figure 2 demonstrate the FTIR spectrum and the NMR spectrum of a PEG-1000 macro crosslinker, respectively.

The IR (cm^−1^); 1723.03 (C=O), 1560.04 (C=C), 1103.76 (C–O–C).

NMR (δ, ppm); 2.0 (CH_3_), 3.5 (–CH_2_–O–CH_2_), 5.6–6.2 (vinylic protons).

#### 2.2.2. Synthesis of the Crosslinked PS-b-PEG Block Copolymers

In our work, the crosslinked PS-b-PEG block copolymers were constructed from styrene copolymerization by the PEG-DM macro crosslinkers by using emulsion polymerization under vacuum in the Schlenk system [52,53,54,55]. M_ω_ values of polyethyleneglycol of PEG were 1000 Da, 1500 Da, and 10,000 Da.

**Scheme 2:**
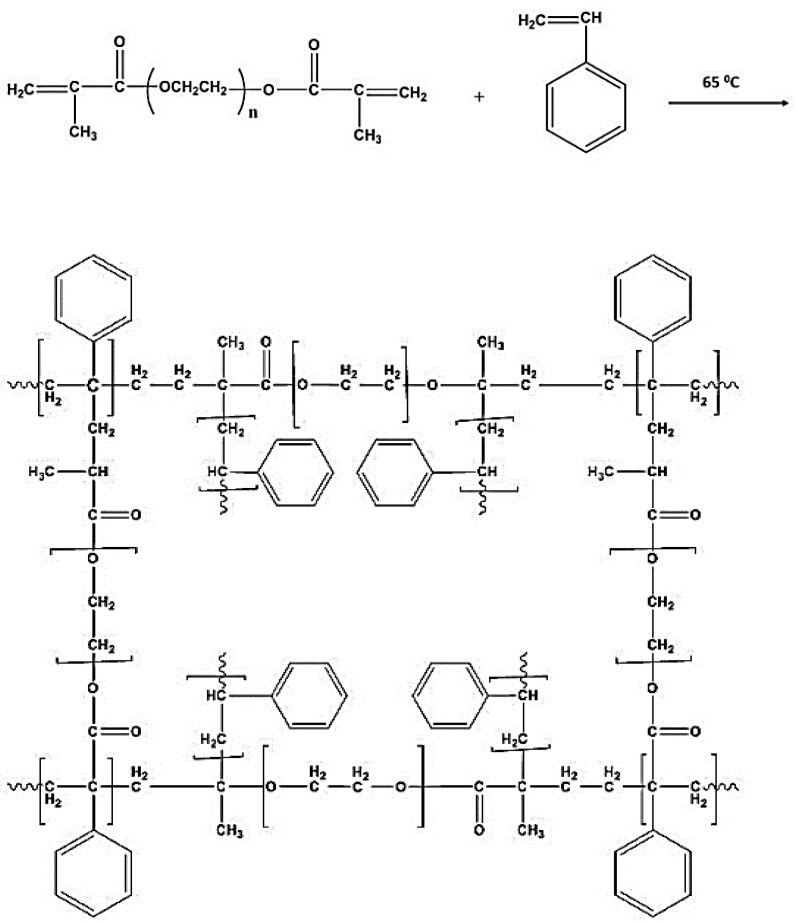
The reaction mechanism of the crosslinked PS-b-PEG Block Copolymer.

**Figure 3:**
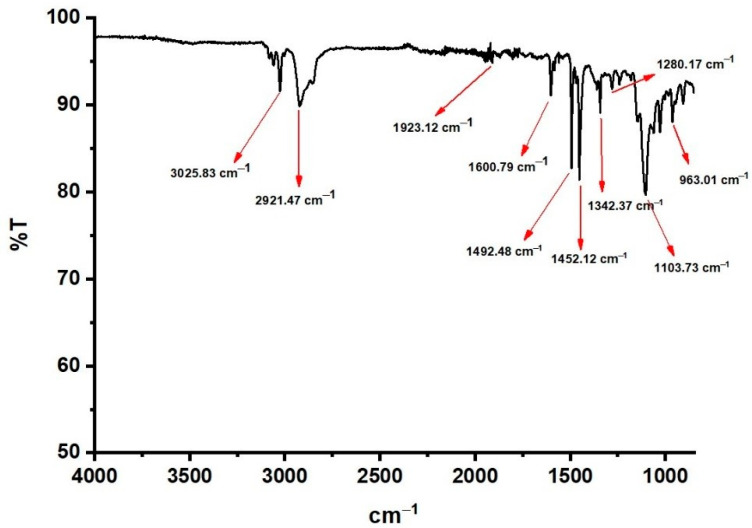
The FTIR spectrum of the PS-b-PEG Block Copolymer acquired by the S polymerization with PEG Macro Crosslinker.

The synthesis process of the crosslinked PS-PEG block copolymers is as follows: 0.768 g of CaHPO_4_2H_2_O, 0.05 g of gelatin, 0.05 g of AIBN, placed in a three-necked balloon into the Schlenk flask, were mixed in 90 mL of deionized water under N_2_ atmosphere for 45 min, the temperature was increased to 65 °C. 10.5 mL of styrene and 2.0242 g of PEG-DM 1000 or PEG-DM 1500 were added to the solution dropwise at the same time in separate dropping funnels. After 5 h, the particulate structure was obtained. The reaction mixture was kept in cold water overnight and then filtered. The crosslinked PS-PEG block copolymer was washed with deionized water and methanol. It was dried in a vacuum oven until constant weight. The reaction pathway of PS-PEG Block Copolymer can be illustrated in Scheme 2. Figure 3 shows the FTIR spectrum of PS-PEG Block Copolymer.

The IR peaks as cm^−1^ in the FTIR spectrum of the crosslinked PS-b-PEG block copolymers are followed: methylene group CH stretching vibrations at 2921.47, benzene ring C=C stretching vibrations at 1600.79, benzene ring overtone band combinations at 1600.79 and 1452.12, –C (C=O) stretching vibrations at 1280.17, and –OCC groups asymmetric stretching vibrations at 1103.73 (in Figure 3).

### 2.3. Characterization

#### 2.3.1. FTIR Spectra

FTIR spectrum of the PEG-1000 or the PEG-1500 and the PEG-10,000 macro crosslinker and the crosslinked PS-b-PEG Block Copolymers were enrolled on a Perkin-Elmer 1600 at room temperature. Dried samples (20 mg) were blended with 100 mg of dry KBr and pressed into disks (100 kg.cm^−2^). The wavenumber range is 580–4000 cm^−1^, and the scan rate is 4 times per second [52,53].

#### 2.3.2. ^1^H NMR Spectra

Varian/Mercury-400 NMR spectrometer was utilized to acquire the ^1^H NMR spectrum of the PEG-1000 or PEG-1500 macro crosslinker. The CDCl_3_ solvent and tetramethylsilane as an internal standard were used.

#### 2.3.3. Thermo Gravimetric Analysis (TGA)

The Thermo Gravimetric Analysis (TGA) of the PEG-1000 or the PEG-1500 and the PEG-10,000 macro crosslinkers and the PS-PEG Block Copolymers was performed using the Seiko II Exstar 6000 analyzer. The PEG-1000, PEG-1500, or the PEG-10,000 macro crosslinkers and the PS-PEG Block Copolymers were investigated using N_2_ atmosphere (200 mL/min) within the temperature intervals 30–500 °C. A heating rate of 20 °C/min was used for this purpose [52,53].

#### 2.3.4. Scanning Electron Micrographs (SEM)

SEM measurements were conducted by Jeol JXA-840 brand electron microscopy. The PEG-1000 or PEG-1500 macro crosslinker and the PS-PEG Block Copolymers were frozen under liquid nitrogen; after that, they were cracked, assembled, and covered with gold (300 Angstrom) on an Edwards S 150 B sputter coater. SEM measurements were fulfilled at 10 kV. Resolution of SEM 3.0 nm at 30 kV in high vacuum, low vacuum, and ESEM. Standard detectors are ETD, low-vacuum SED (LVD), gaseous SED for ESEM mode (GSED), IR camera. Electron images were enrolled directly on a Polaroid film from the cathode ray tube. The enlargement in the measurements is from 1000× up to 15,000× [52,53].

### 2.4. Preparation of the PbO Doped the Crosslinked PS-b-PEG Block Copolymers and the PbO Doped the PS-b-PEG-BN Nanocomposite Materials

Table 1 represents the construction of each of the explored substances. The tablets of our composites were produced at 22 °C room temperature, handling a hydraulic compression under 10 MPa stress for 20 min. The tablets have a diameter of 12 mm and their thickness was measured using a BTS 12051 µm (0–25 MMX 0.01 mm).

### 2.5. Gamma Ray Attenuation Measurements

The gamma irradiation attenuation factors of the investigated composites were acquired for wide-range energy spectrums, emitted from E 152u source using an HPGe (High Purity Germanium) detector framework at Karadeniz Technical University, Faculty of Science, Department of Physics. The model of the experimental detecting geometry is illustrated in Figure S1 (the figure is available in Supplementary information file; Figure S1). To acquire experimental outputs throughout the surveying procedure benefited from the Gamma Vision (Version: 6.07-Ortec, Oak Ridge-Tennessee-USA) computer program that maintains sophisticated multi-channel analyzer capabilities.

## 3. Results and Discussions

### 3.1. Constructional Information of Samples

#### 3.1.1. Thermogravimetric Analysis (TGA) of the PbO Doped the Crosslinked PS-b-PEG Block Copolymers and the PbO Doped the PS-b-PEG Nanocomposite Materials

**Figure 4:**
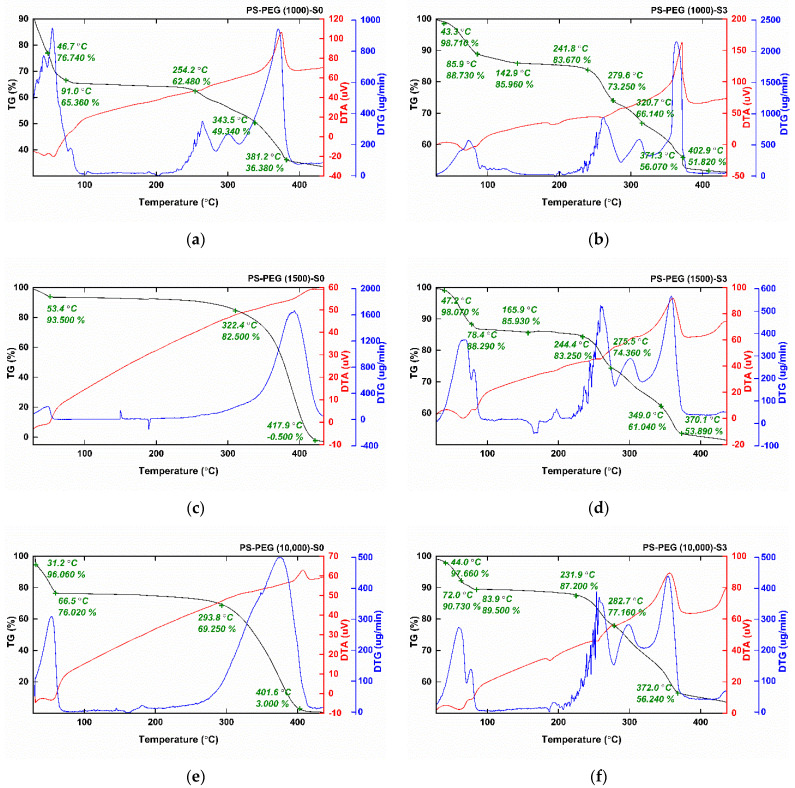
TGA thermograms of the PbO doped the crosslinked PS-b-PEG block copolymers materials: (**a**) PS-PEG (1000)-S0; (**b**) PS-PEG (1000)-S3; (**c**) PS-PEG (1500)-S0; (**d**) PS-PEG (1500)-S3; (**e**) PS-PEG (10,000)-S0; (**f**) PS-PEG (10,000)-S3. Black, blue and red lines indicate the thermogravimetry (TG), derivative thermogravimetry (DTG), and differential thermal analysis (DTA) values respectively.

**Figure 5:**
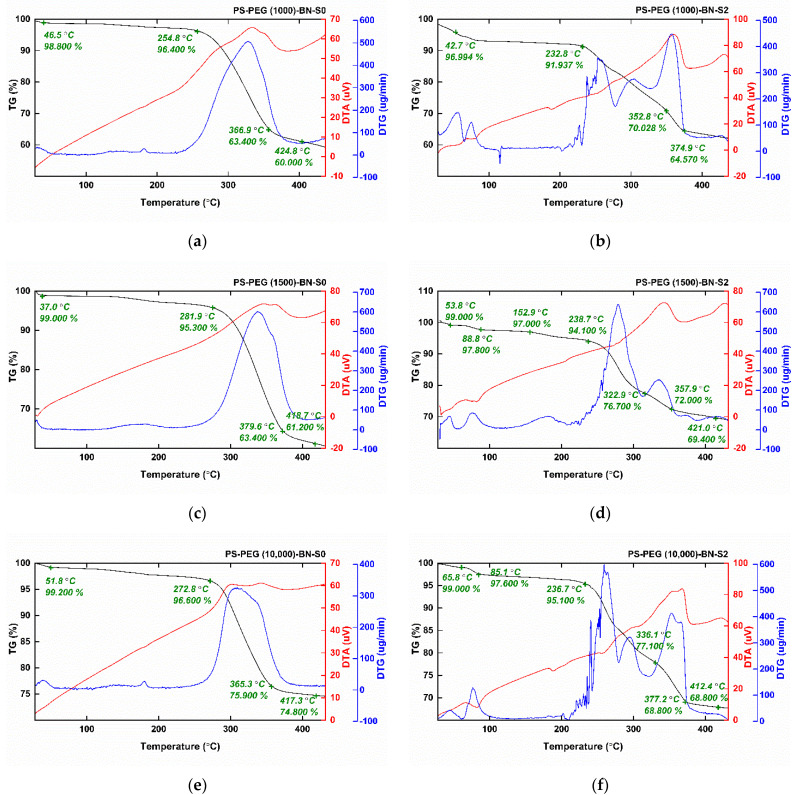
TGA thermograms of the PbO doped the PS-b-PEG-BN nanocomposite materials: (**a**) PS-PEG (1000)-BN-S0; (**b**) PS-PEG (1000)-BN-S2; (**c**) PS-PEG (1500)-BN-S0; (**d**) PS-PEG (1500)-BN-S2; (**e**) PS-PEG (10,000)-BN-S0; (**f**) PS-PEG (10,000)-BN-S2. Black, blue and red lines indicate the thermogravimetry (TG), derivative thermogravimetry (DTG), and differential thermal analysis (DTA) values respectively.

TGA thermograms of the PbO doped the crosslinked PS-b-PEG block copolymers and the PbO doped the PS-b-PEG-BN nanocomposite materials are given in Figure 4 and Figure 5, and Table 2. TGA thermograms in the figure belong to these samples containing PS-PEG (1000)-S0, PS-PEG (1000)-S3, PS-PEG (1500)-S0, PS-PEG (1500)-S3, PEG (10,000)-S0, PS-PEG (10,000)-S3, PS-PEG (1000)-BN-S0, PS-PEG (1000)-BN-S2, PS-PEG (1500)-BN-S0, PS-PEG (1500)-BN-S2, PEG (10,000)-BN-S0, and PS-PEG (10,000)-BN-S2, respectively, the contents of the samples were given in Table 1.

As can be viewed from the thermograms in Figure 4a, the temperature at which the PS-PEG (1000)-S0 sample containing 100% PS-PEG (1000) primary starts to degrade is 46.7 °C and there is an 11.380% decrement among 46.7–91.0 °C. The genuine deterioration starts at 254.2 °C and 36.380% declines at 381.2 °C. The PS-PEG (1000)-S3 sample containing 10% PS-PEG (1000) and 90% lead-oxide in Figure 4b, 1.290% of the composites commenced to deteriorate at 43.3 °C and there is a 9.980% decrement among 43.3–85.9 °C. The amount nearly remaining among 85.9–241.8 °C is read from the thermogram as 88.730–83.670%. It began to alter from 83.670% at 241.8 °C to 56.070% at 371.3 °C. Furthermore, there is a 31.850% decrement among 241.8–402.9 °C.

In Figure 4c, the temperature at which the PS-PEG (1500)-S0 sample containing 100% PS-PEG (1500) primary starts to degrade is 53.4°. The amount nearly remaining among 53.4–322.4 °C is read from the thermogram as 93.500–82.500%. It began to alter from 82.500% at 322.4 °C to 0.500% at 417.9 °C. The temperature at which the PS-PEG (1500)-S3 sample containing 10% PS-PEG (1500) and 90% lead-oxide in Figure 4d primary starts to degrade is 47.2 °C and there is a 9.780% decrement among 47.2–78.4 °C. The amount nearly remaining among 78.4–244.4 °C is read from the thermogram as 88.290–83.250%. The genuine deterioration starts at 244.4 °C and 53.890% declines at 370.1 °C.

In Figure 4e, the temperature at which the PS-PEG (10,000)-S0 sample containing 100% PS-PEG (10,000) primary starts to degrade is 31.2 °C and there is a 20.040% decrement among 31.2–66.5 °C. The amount nearly remaining among 66.5–293.8 °C is read from the thermogram as 76.020–69.250%. It began to alter from 69.250% at 293.8°C to 3.000% at 401.6 °C, there is a 66.250% decrement among 293.8–401.6 °C. The temperature at which the PS-PEG (10,000)-S3 sample containing 10% PS-PEG (10,000) and 90% lead-oxide (in Figure 4f) primary starts to degrade is 44.0 °C and there is an 8.160% decrement among 44.0–83.9 °C. The amount nearly remaining among 83.9–231.9 °C is read from the thermogram as 89.500–87.200%. The genuine deterioration starts at 231.9 °C and 56.240% declines at 372.0 °C. There is a 30.960% decrement among 231.9–372.0 °C.

As can be observed from the thermograms in Figure 5a, the temperature at which the PS-PEG (1000)-BN-S0 sample containing 50% PS-PEG (1000) and 50% boron nitride primary starts to degrade is 46.5°C. The amount nearly remaining among 46.5–254.8 °C is read from the thermogram as 98.800–96.400%. The genuine deterioration starts at 254.8 °C and 63.400% declines at 366.9 °C. There is a 36.400% decrement among 254.8–424.8 °C. The PS-PEG (1000)-BN-S2 sample containing 5% PS-PEG (1000), 5% boron nitride, and 90% lead-oxide in Figure 5b, 3.006% of the composites commenced to deteriorate at 42.7 °C. The amount nearly remaining among 42.7–232.8 °C is read from the thermogram as 96.994–91.937%. It began to alter from 91.937% at 232.8 °C to 70.028% at 352.8 °C. Furthermore, there is a 27.367% decrement among 232.8–374.9 °C.

In Figure 5c, the temperature at which the PS-PEG (1500)-BN-S0 sample containing 50% PS-PEG (1500) and 50% BN primary starts to degrade is 37.0°C and there is a 3.700% decrement among 37.0–281.9 °C. The genuine deterioration starts at 281.9 °C and 63.400% declines at 379.6 °C. There is a 34.100% decrement among 281.9–418.7 °C. The PS-PEG (1500)-BN-S2 sample containing 5% PS-PEG (1500), 5% BN, and 90% PbO in Figure 5d, 1.000% of the composites commenced to deteriorate at 53.8 °C. The amount nearly remaining among 88.8–238.7 °C is read from the thermogram as 97.800–94.100%. It began to alter from 94.100% at 238.7 °C to 76.700% at 322.9 °C. Furthermore, there is a 24.700% decrement among 238.7–421.0 °C.

The temperature at which the PS-PEG (10,000)-BN-S0 sample containing 50% PS-PEG (10,000) and 50% BN primary starts to degrade is 51.8 °C and there is a 2.600% decrement among 51.8–272.8 °C in Figure 5e. The genuine deterioration starts at 272.8 °C and 75.900% declines at 365.3 °C. There is a 21.800% decrement among 272.8–417.3 °C. The PS-PEG (10,000)-BN-S2 sample containing 5% PS-PEG (10,000), 5% BN, and 90% PbO in Figure 5f, 1.000% of the composites commenced to deteriorate at 65.8 °C. The amount nearly remaining among 85.1–236.7 °C is read from the thermogram as 97.600–95.100%. It began to alter from 95.100% at 236.7 °C to 77.100% at 336.1 °C and there is an 18.000% decrement among these temperatures. There is an 8.300% decrement among 336.1–412.4 °C. Furthermore, there is a 26.300% decrement among 236.7–412.4 °C.

When we examine the outcomes of TGA thermograms of the PbO doped the crosslinked PS-b-PEG block copolymers and the PbO doped the PS-b-PEG-BN nanocomposite materials, it is seen that the implemented radiation does not influence the structure of the research sample.

While the initial decomposition temperature of the copolymer (PS-PEG (1000)-S0) was 46.7 °C and the amount of copolymer remaining without decomposition was 76.740% (in Figure 4a) when 90% PbO was added to the copolymer (PS-PEG (1000)-S3), the initial decomposition temperature decreased by 3.4 °C, but the amount of composite remaining without decomposition increased (in Figure 4b). Even when the PS-PEG (1000)-S3 composite is at 402.9 °C, the amount of composite remaining without deterioration is 51.820%. This shows that PbO increases the durability of the copolymer.

The initial decomposition temperature of PS-PEG (1000)-BN-S0 composite with 50% BN added to 50% polymer is close to the initial decomposition temperature of PS-PEG (1000)-S0 copolymer and it was observed that the amount of composite remaining without decomposition was 98.800% (in Figure 5a). In addition, the amount of composite remaining without decomposition at 424.8 °C is 60%. This indicates that BN increases the thermal stability of the copolymer.

The initial decomposition temperature of PS-PEG (1000)-BN-S2 composite formed by the addition of 90% PbO and 5% BN is 42.7 °C and the amount of composite remaining without decomposition is 96.994% (in Figure 5b). It was observed that there was not much change in the amount of composite that remained intact as the temperature increased. The amount of composite remaining without decomposition at 374.9 °C is 64.570%.

The initial decomposition temperature of PS-PEG (1500)-S0 copolymer is 53.4 °C and the amount of copolymer remaining without decomposition is 93.500%. At 417.9 °C the copolymer completely decomposed (in Figure 4c).

Although the initial decomposition temperature of the PS-PEG (1500)-S3 composite with 90% PbO is below the initial decomposition temperature of the PS-PEG (1500)-S0 copolymer, the amount of composite remaining without decomposition is 98.070 (in Figure 4d). As the temperature increases, the percentage of decomposition increases, but at 370.1 °C the amount of composite remaining without decomposition is 53.890%. In other words, PbO increased the thermal stability of the copolymer.

When 50% BN was added to the copolymer, the initial decomposition temperature of the PS-PEG (1500)-BN-S0 composite decreased, but the amount of decomposed composite decreased (in Figure 5c). Although the PS-PEG (1500)-S0 copolymer completely decomposes at 417.9 °C, the amount of composite remaining without decomposition at 418.7 °C with the addition of BN is 61.200%. It was observed that boron nitride increased the thermal stability of the copolymer.

The initial decomposition temperature (53.8 °C) of PS-PEG (1500)-BN-S2 copolymer, in which 5% BN and 90% PbO are added, is slightly higher than PS-PEG (1500)-S0 copolymer and the remaining composite amount without decomposition is 99% (in Figure 5d). As can be seen from the thermogram, when BN and PbO were added to the copolymer, which decomposed completely at 417.9 °C, 69.400% of the nanocomposite remained intact at 421.0 °C.

The initial decomposition temperature of PS-PEG (10,000)-S0 copolymer is 31.2 °C and the remaining copolymer amount is 96.060%. The copolymer decomposed 97% at 401.6 °C (in Figure 4e).

When 90% PbO is added to PS-PEG (10,000)-S0 copolymer, the initial decomposition temperature of the nanocomposite formed is 2.8 °C higher than the decomposition temperature of the copolymer, and the remaining composite amount without decomposition is 97.660% (in Figure 4f).

The initial deterioration temperature and the remaining composite amount without decomposition of PS-PEG (10,000)-BN-S0 composite formed when 50% BN is added to PS-PEG (10,000)-S0 copolymer is higher than PS-PEG (10,000)-S0 and PS-PEG (10,000)-S3 (in Figure 5e). While 75.900% of the PS-PEG (10,000)-BN-S0 composite remains intact at 356.3 °C, the remaining composite amount of the PbO added composite (PS-PEG (10,000)-S3) is 56.240% at 372.0 °C. Considering these values, the BN doped nanocomposite has higher thermal stability than both the copolymer and PbO doped nanocomposites.

The initial decomposition temperature of PS-PEG (10,000)-BN-S2 copolymer containing 5% BN and 90% PbO was higher than its other counterparts and decomposed at 1% (in Figure 5f). BN and PbO increased the thermal stability of the copolymer.

As the molecular weight of the PEG crosslinker in the PS-PEG copolymer increased, the deterioration percentage of the copolymer increased from 93.500% to 96.060%. The final decomposition temperature varies among 381.2 °C and 417.9 °C.

#### 3.1.2. Morphological Characterization of the PbO Doped the Crosslinked PS-b-PEG Block Copolymers and the PbO Doped the PS-b-PEG Nanocomposite Materials

The morphological characterization of the samples in this research was performed via ZEISS EVO LS 10 (Carl Zeiss NTS, Germany) scanning electron microscope in the Central Research Laboratory at Karadeniz Technical University. Information on topography, morphological analysis, internal structure analysis (phase, grain size, grain shape, etc.), fracture surface and crack analysis, and surface element analysis (composition) of the samples were obtained (all SEM images are available in Supplementary information file; Figures S2–S13). Furthermore, high-resolution images were also obtained with Secondary Electron imaging (SE) and Back-Reflected Electron (BSE) detectors in the device.

Figure S2 shows SEM photographs of the crosslinked PS-b-PEG (1000) block copolymer. As seen from the SEM films, the crosslinked PS-b-PEG (1000) block copolymer exhibits a surface appearance containing particles, layers, pores and branchings, and voids (in Figure S2a,b). A homogeneous continuous matrix was observed in which PS and PEG units were interconnected. The surface of the polymer is granular (in Figure S2a) and porous (in Figure S2b). PS blocks formed a continuous phase and a branched, rough surface (in Figure S2b) with macro crosslinker PEG (1000) (in Figure S2a). Micrometer and nanometer particle sizes were measured for crosslinked PS-b-PEG block copolymers. Measured particle sizes were 2.262, 2.316 µm (in Figure S2a) and 429.0, 478.4, 485.2 nm (in Figure S2b).

SEM photographs of the PS-b-PEG (1500) block copolymer are presented in Figure S3a,b. As seen in Figure S3, there are fractures (in Figure S3b), cracks (in Figure S3a,b), and smooth (in Figure S3a) parts on the surface of the crosslinked block copolymer.

Figure S4a,b shows SEM photographs of the crosslinked PS-b-PEG (10,000) block copolymer. There are smooth regions (in Figure S4b) as well as branches, particles (in Figure S4a), voids (in Figure S4a,b), and pores (in Figure S4a) on the surface of the polymer. Although the particle sizes for the crosslinked PS-b-PEG (1000) block copolymer are 2.262 µm, 2.316 µm and 429.0 nm, 478.4 nm, and 485.2 nm; the particle sizes for the crosslinked PS-b-PEG (10,000) block copolymer are measured as 6.997 µm, 9.334 µm. As the molecular weight of the crosslinker PEG increases, the polymer particle size increases.

In general, although the morphologies of the three crosslinked block copolymers examined were similar to each other, it was observed that as the molecular weight of the PEG macro crosslinker increased, the particulate structure decreased, and there was aggregation and branching on the surface.

The crosslinked PS-b-PEG (1000) block copolymer was homogeneously mixed with the same amount of BN nanoparticles, and it was observed that the morphology of the crosslinked PS-b-PEG (1000) block copolymer-BN nanocomposite was similar to the morphology of the crosslinked PS-b-PEG (1000) block copolymer (see in Figure S5a,b). A homogeneous structure was formed as a result of the penetration of 1 µm sized BN nanoparticles into the crosslinked PS copolymer pores. In Figure S5a, the polymer particle size was measured as 3.089 µm.

The SEM images of the crosslinked PS-b-PEG (1500) block copolymer+BN nanocomposite are similar to the images of the PS-b-PEG (1500) block copolymer+BN nanocomposite in Figure S6. BN nanoparticles mixed homogeneously with PS-b-PEG (1500) block copolymer. As seen in Figure S6a,b, there are roughnesses, particles, pores, and elevations on the surface.

Figure S7 exhibits SEM photographs of the crosslinked PS-b-PEG (10,000) block copolymer-BN nanocomposite (50% PS-PEG (10,000)+50% BN+0% PbO in Table 1). As seen in Figures S5 and S6, the surface morphology of the crosslinked PS-b-PEG (10,000) block copolymer+BN nanocomposite is similar to that of the PS-b-PEG (1000) block copolymer+BN nanocomposite and the crosslinked PS-b-PEG (1500) block copolymer+BN nanocomposites. The molecular size and the crack distance of the crosslinked PS-b-PEG (10,000) block copolymer+BN nanocomposite are 3.682 µm and 19.500 µm (in Figure S7b).

In 10% PS-PEG (1000)+0% BN+90% PbO composite, in which 90% lead oxide is added into 10% polymer, the crosslinked PS-b-PEG (1000) block copolymer surface is rough (in Figure S8a), granular (in Figure S8b), as in other examples. It is observed that there are porous (in Figure S8b), voids (in Figure S8a,b) and clusters (in Figure S8b) as seen from the SEM photograph in Figure S8a, the size of the PbO particle on the polymer matrix surface is 681.6 nm.

Figure S9a,b shows SEM photographs of the crosslinked PS-b-PEG (1000) block copolymer+BN+PbO nanocomposite. It was observed that the surface morphology of the 5% BN and 90% lead oxide doped, 5% PS-PEG (1000)+5% BN+90% PbO composite was similar as seen from the SEM photographs of the crosslinked PS-b-PEG (1000) block copolymer+PbO nanocomposite (10% PS-PEG (1000)+0% BN+90% PbO in Table 1, in Figure S8). The crosslinked PS-b-PEG (1000) block copolymer+BN+PbO nanocomposite surface is rough (in Figure S9a), granular (in Figure S9a), porous (in Figure S9a,b), and has gaps (in Figure S9b) and clusters (in Figure S9b). As can be seen from the SEM photograph of Figure S9b, the dimensions of the PbO and BN particles on the polymer surface are 272.6 nm, 424.2 nm. It was observed that the dimensions of the PbO on the polymer surface are 681.6 nm in Figure S8a. Figure S10a,b shows SEM photographs of the crosslinked PS-b-PEG (1500) block copolymer+PbO nanocomposite (10% PS-PEG (1500)+0% BN+90% PbO in Table 1). SEM images of crosslinked PS-b-PEG (1500) block copolymer+PbO nanocomposite are similar to SEM images of (10% PS-PEG (1000)+0% BN+90% PbO) and (10% PS-PEG (10,000)+0% BN+90% PbO) composites. In the surface morphology of this composite, there are rough (in Figure S10b) and smooth surfaces (in Figure S10a), particles (in Figure S10b), voids (in Figure S10a,b), pores (in Figure S10b) and particle clusters (in Figure S10a,b) as seen in the surface films of other composites. Figure S10a shows that the particle size on the composite surface is 500.9 nm and 512.7 nm.

SEM photographs of the crosslinked PS-b-PEG (1000) block copolymer+BN+PbO nanocomposite were presented in Figure S11a,b. The surface morphology of 5% PS-PEG (1500)+5% BN+90% PbO nanocomposite is similar to the surface morphology of 5% PS-PEG (1000)+5% BN+90% PbO composite (in Figure S9). There are roughnesses (in Figure S11a,b), pores (in Figure S11a,b), particles (in Figure S11b), fractures (in Figure S11a,b) on the surface of 5% PS-PEG (1500)+5% BN+90% PbO nanocomposite.

Figure S12a,b shows SEM photographs of the crosslinked PS-b-PEG (10,000) block copolymer+PbO nanocomposite (10% PS-PEG (10,000)+0% BN+90% PbO in Table 1). The surface morphology of the crosslinked PS-b-PEG (10,000) block copolymer+PbO nanocomposite is similar to the surface morphology of the crosslinked PS-b-PEG (1000) block copolymer (100% PS-PEG (10,000)+0% BN+0% PbO in Table 1) and the crosslinked PS-b-PEG (10,000) block copolymer (100% PS-PEG (10,000)+0% BN+0% PbO in Table 1). In the SEM photographs in Figure S12a,b, there are roughnesses (in Figure S12a,b), clustering of polymer particles (in Figure S12a), voids (in Figure S12a), and pores (in Figure S12b) on the surface. The particle size of the crosslinked PS-b-PEG (10,000) block copolymer+PbO nanocomposite is 972.2 nm and 1.088 µm (in Figure S12b).

SEM photographs of the crosslinked PS-b-PEG (10,000) block copolymer+BN+PbO nanocomposite are in Figure S13a,b. The surface morphology of the crosslinked PS-b-PEG (10,000) block copolymer+BN+PbO nanocomposite is similar as seen from the SEM photographs of the other composites. There are particles (in Figure S13b), roughness (in Figure S13b), pores (in Figure S13a,b), voids (in Figure S13b), rough and smooth parts (in Figure S13a) on the surface of the composite.

### 3.2. Gamma-Ray Attenuation Characteristics of the PbO Doped the Crosslinked PS-b-PEG Block Copolymers and the PbO Doped the PS-b-PEG-BN Nanocomposite Materials

#### 3.2.1. Linear ($\mu_{linear}$) and Mass Attenuation ($\mu_{mass}$) Coefficients

**Figure 6:**
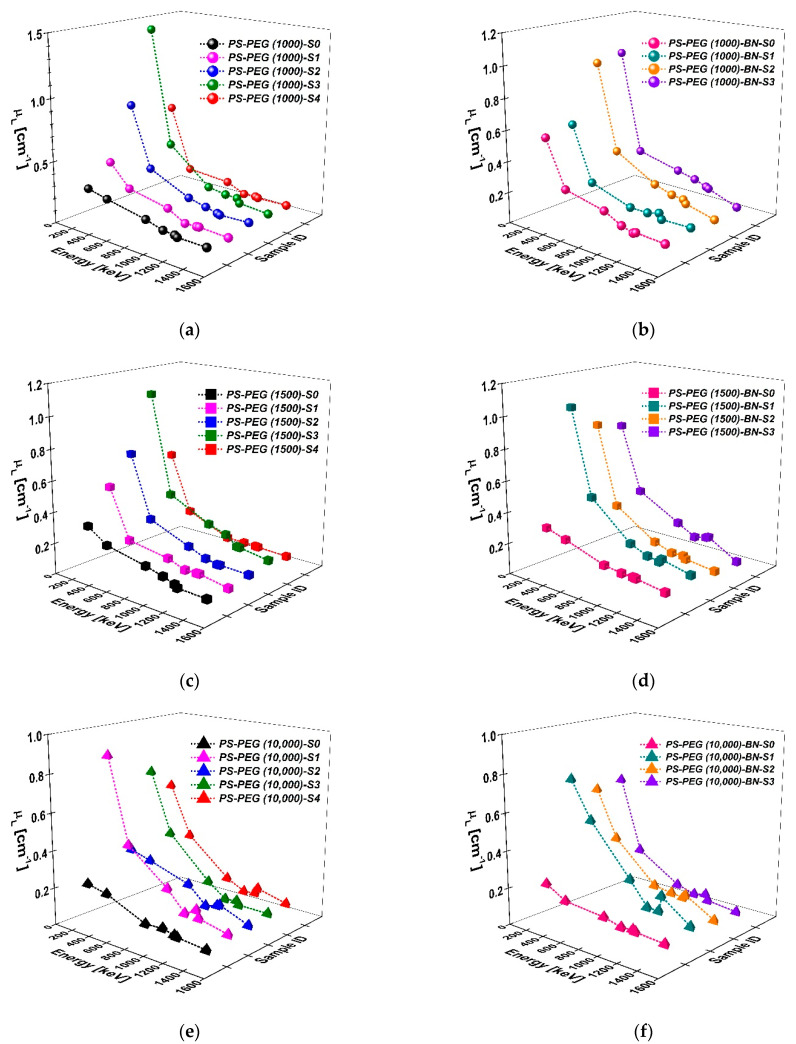
The LACs values of the PbO doped the crosslinked PS-b-PEG block copolymers and the PbO doped the PS-b-PEG-BN nanocomposite materials at different gamma radiation energies (from 121.782 keV to 1408.006 keV) of E 152u: (**a**) PS-PEG (1000)-S0,S1,S2,S3,S4; (**b**) PS-PEG (1000)-BN-S0,S1,S2,S3; (**c**) PS-PEG (1500)-S0,S1,S2,S3,S4; (**d**) PS-PEG (1500)-BN-S0,S1,S2,S3; (**e**) PS-PEG (10,000)-S0,S1,S2,S3,S4; (**f**) PS-PEG (10,000)-BN-S0,S1,S2,S3.

The Linear Attenuation Coefficients (LACs) at the 121.782 keV, 344.279 keV, 778.904 keV, 964.079 keV, 1085.869 keV, 1112.074 keV and 1408.006 keV gamma-ray energy intervals are illustrated in Figure 6 and Tables S1 and S2 (tables are available in Supplementary information file; Tables S1 and S2). It is clear that the attenuation aptitude little by little decreases for all samples in regions where the gamma-ray energy ascends. Namely, it is more difficult for shielding photons in the high gamma energies than in the low gamma energies of photons.

There was admissible consistency observed between the experimental and theoretical LACs of investigated samples. An ascent in gamma rays shielding effectiveness as delineated in Figure 6 is pronounced for our samples. Thus, the mixing of PbO in the crosslinked PS-b-PEG block copolymers and the PS-b-PEG-BN nanocomposites matrix outcomes in an ascent in the odds of interaction between the incoming gamma irradiation and the shielding material atoms. It can be deduced that our samples can treat as shieldings also opposed to the low dose proportions from gamma irradiation origins.

The LACs values decrease along with a swell in gamma radiation energies (as can be observed in Figure 6. The occasion for this situation is the interaction of gamma radiation with materials via a photoelectric effect, Compton scattering, and pair production. The photoelectric effect is most influential in the low gamma energy regions; for this case, LACs values are greater in these gamma radiation energy areas. Compton effect is overpowering at middle radiation energy areas, conversely, pair production is dominant at elevated gamma irradiation energy regions; herewith, LACs worthies start to reduce with the escalate of gamma energies.

As it is clearly emphasized in Tables S1 and S2, and Figure 6a–f, it has been observed that the measured and calculated values show changes with the change of the polymer type used to improve the gamma radiation shielding materials. In addition, it was observed that the radiation protection capacities of the samples improved when the lead oxide or boron nitride percentages of the prepared materials were changed. When we compare all samples, we can deduce that the higher LAC worthies and best absorption of photons are owing to the high percentage rates of PbO ingredients in the substances. If the LACs results of our all samples are examined with a broad perspective, it can be emphasized that these samples developed in our research reveal important and reliable results to enlighten radiation shielding studies.

**Figure 7:**
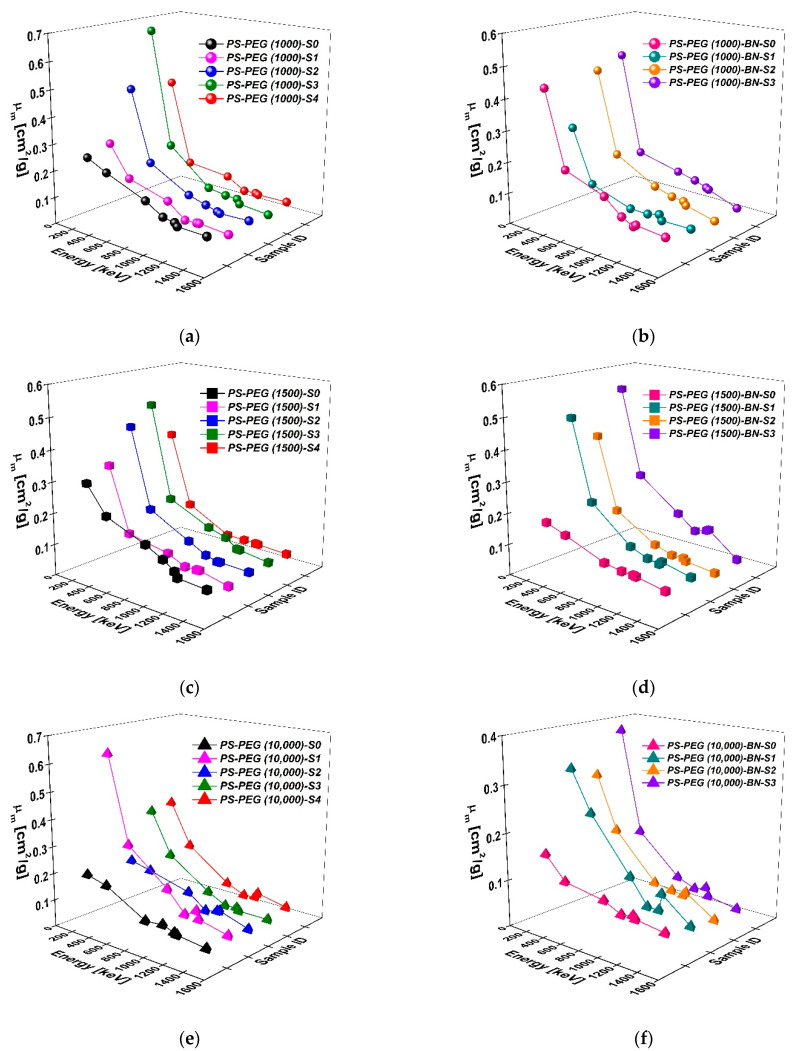
The MACs values of the PbO doped the crosslinked PS-b-PEG block copolymers and the PbO doped the PS-b-PEG-BN nanocomposite materials at different gamma radiation energies (from 121.782 keV to 1408.006 keV) of E 152u: (**a**) PS-PEG (1000)-S0,S1,S2,S3,S4; (**b**) PS-PEG (1000)-BN-S0,S1,S2,S3; (**c**) PS-PEG (1500)-S0,S1,S2,S3,S4; (**d**) PS-PEG (1500)-BN-S0,S1,S2,S3; (**e**) PS-PEG (10,000)-S0,S1,S2,S3,S4; (**f**) PS-PEG (10,000)-BN-S0,S1,S2,S3.

The Mass Attenuation Coefficients (MACs) at the 121.782 keV, 344.279 keV, 778.904 keV, 964.079 keV, 1085.869 keV, 1112.074 keV, and 1408.006 keV gamma-ray energy intervals are illustrated in Figure 7 and Tables S3–S4 (tables are available in Supplementary information file; Tables S3 and S4). It is clear that the attenuation aptitude little by little decreases for all samples in regions where the gamma-ray energy ascends. Namely, it is more difficult to shielding photons in the high gamma energies than in the low gamma energies of photons.

There was admissible consistency observed between the experimental and theoretical MACs of investigated samples. An ascent in shielding effectiveness as demonstrated in Figure 7 is evident for our samples. Thus, the mixing of PbO in the crosslinked PS-b-PEG block copolymers and the PS-b-PEG-BN nanocomposites matrix outcomes in an ascent in the possibility of interaction among the incoming gamma radiation and the gamma rays shielding atoms. It can be deduced that our samples can treat as shields also against low dose rates from gamma irradiation sources. The MACs values decrease along with a swell in gamma radiation energies (observed in Figure 7). The occasion for this situation is the interaction of gamma radiation with materials via a photoelectric effect, Compton scattering, and pair production. The photoelectric effect is most influential in the low gamma energy regions; for this case, MACs values are greater in these gamma radiation energy areas. Compton effect is overpowering at middle radiation energy areas, conversely, pair production is dominant at elevated gamma irradiation energy regions; herewith, MACs worthies start to reduce with the escalate of gamma energies.

As it is clearly emphasized in Tables S3 and S4, and Figure 7a–f, it has been observed that the measured and calculated values show changes with the change of the polymer type used to improve the gamma radiation shielding materials. In addition, it was observed that the radiation protection capacities of the samples improved when the PbO or BN percentages of the prepared materials were changed. When we compare all samples, we can deduce that the bigger MACs and ideally absorption of gamma photons are owing to the elevated percent rates of PbO ingredient in the substances. If the MACs results of all our samples are examined with a broad perspective, it can be emphasized that these samples developed in our research reveal important and reliable results to enlighten radiation shielding studies.

As seen in Figure 6a–f and Tables S1 and S2, $\mu_{L}$ values decrease as energy values increase. In the energy interval of 121.788–1408.006 keV, the order of $\mu_{L}$ values for PS-PEG (1000) copolymer and its composites are PS-PEG (1000)-S0<PS-PEG (1000)-S1<PS-PEG (1000)-S4<PS-PEG (1000)-S2<PS-PEG (1000)-S3. The order of $\mu_{L}$ values for PS-PEG (1000) composites containing PbO and BN is PS-PEG (1000)-BN-S0<PS-PEG (1000)-BN-S1<PS-PEG (1000)-BN-S2<PS-PEG (1000)-BN-S3. The order of $\mu_{L}$ values of PS-PEG (1500), PS-PEG (10,000), and composites of these copolymers are in the same order as PS-PEG (1000) copolymer and composites. Furthermore, as seen in Figure 7a–f and Tables S3 and S4, $\mu_{m}$ values decrease as energy values increase. In the energy interval of 121.788 keV-1408.006 keV, the order of $\mu_{m}$ values for PS-PEG (1500) copolymer and its composites are PS-PEG (1500)-S0<PS-PEG (1500)-S1<PS-PEG (1500)-S4<PS-PEG (1500)-S2<PS-PEG (1500)-S3. The order of $\mu_{m}$ values for PS-PEG (1500) composites containing PbO and BN is PS-PEG (1500)-BN-S0<PS-PEG (1500)-BN-S2<PS-PEG (1500)-BN-S1<PS-PEG (1500)-BN-S3. The order of $\mu_{m}$ values of PS-PEG (1000), PS-PEG (10,000), and composites of these copolymers are in the same order as PS-PEG (1500) copolymer and composites.

We can see from the PS-PEG (10,000)-S0, PS-PEG (1500)-S0, and PS-PEG (1000)-S0 samples that the polymer alone contributes to the absorption. These samples are composed of 100% polymer and when the measurement results are examined, it can be seen that they are useful in radiation absorption. We saw this result when we calculated the theoretical values of the polymers, and we concluded that the experiment and theory agree. We knew from the theoretical results that samples with added PbO would increase the absorption. Absorption increased with increasing PbO additive ratios. PS-PEG (1500)-S1 sample contains 50% PbO. PS-PEG (1500)-S3 sample contains 90% PbO. When the absorption results are examined, PS-PEG (1500)-S3 sample has an average of 31% better absorption value than PS-PEG (1500)-S1 sample, PS-PEG (1000)-S3 (contains 90% PbO) sample, PS-PEG (1000)-S1 (contains 50% PbO) sample increases the absorption by an average of 63%. PS-PEG (10,000)-S3 (contains 90% PbO), PS-PEG (10,000)-S1 (contains 50% PbO) samples stand out as an exception to this situation. Here, we concludes that the PS-PEG (10,000)-S1 sample absorbs on average 41% better than the PS-PEG (10,000)-S3 sample. When we compared this result with theoretical calculations, we saw that the same situation emerged. In this case, it is possible to say that the absorption contribution of PS-PEG (10,000) polymer is stronger than the other two polymers.

When the average values of our BN added samples (PS-PEG (10,000)-BN-S0, PS-PEG (1500)-BN-S0, PS-PEG (1000)-BN-S0) are compared with the ones without boron additives (PS-PEG (10,000)-S0, PS-PEG (1500)-S0, PS-PEG (1000)-S0), the absorption in the undoped samples is 13% for PS-PEG (10,000), 35% for PS-PEG (1500), 7% for PS-PEG (1000), better absorption values are observed. This is also consistent with the theory. Absorption of gamma radiation was not expected from the BN at this point. This result is obvious. It was added because the neutron absorption property of BN is known. It is expected that this important point will be investigated in further studies and its neutron absorber property will be observed.

#### 3.2.2. Half-Value Layer (HVL), Tenth Value Layer (TVL), Mean Free Path (MFP), and Radiation Protection Efficiency (RPE)

The obtained HVL, TVL, and MFP values of these samples are the most crucial indicators of the radiation shielding performance for our developed shielding materials from 121.782 keV to 1408.006 keV gamma-ray energy intervals. The lower the value of these coefficients, the most influential their radiation protection efficiency is. The HVL, TVL, and MFP values scale up or decline in a common characteristic. These values were computed using the Equations (S7), (S8), and (S9) with the help of MACs of all samples for gamma-ray energy regions ranging from 121.782 keV to 1408.006 keV (all equations are available in Supplementary information file; Equations (S1)–(S11). It can be deduced from these figures, the HVL, TVL, and MFP values were altered when the content of the additive materials was altered in the samples. Namely, it can be declared that when the density of the material ascending, the probability of interacting with the gamma-ray photons of the atoms enhancements, causing fewer photons to be transferred from the investigated sample. As a result, the efficiency of the gamma irradiation protection of the sample is contrarily commensurate to the HVL, TVL, and MFP values. The ratios of the HVL, TVL, and MFP values demonstrate a thin layer in the samples in which the strength of gamma radiation can be expressed.

**Figure 8:**
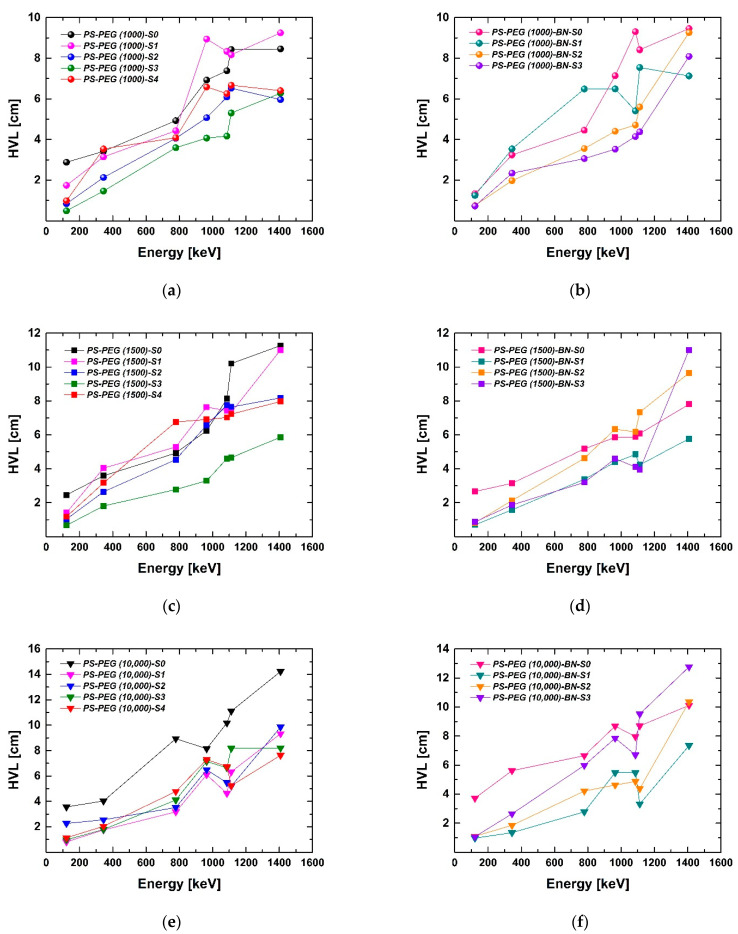
The HVL values of the PbO doped the crosslinked PS-b-PEG block copolymers and the PbO doped the PS-b-PEG-BN nanocomposite materials at different gamma radiation energies (from 121.782 keV to 1408.006 keV) of E 152u: (**a**) PS-PEG (1000)-S0,S1,S2,S3,S4; (**b**) PS-PEG (1000)-BN-S0,S1,S2,S3; (**c**) PS-PEG (1500)-S0,S1,S2,S3,S4; (**d**) PS-PEG (1500)-BN-S0,S1,S2,S3; (**e**) PS-PEG (10,000)-S0,S1,S2,S3,S4; (**f**) PS-PEG (10,000)-BN-S0,S1,S2,S3.

As can be seen in Tables S5 and S6 (tables are available in Supplementary information file; Tables S5 and S6) and Figure 8a–f, the HVL value is the shielding thickness value required to stop half of the emitted photons, that is to absorb them, increases as the photon energy increased. The HVL is a very important marker of the radiation shielding characteristic of the material concerned, i.e., it can be concluded that the lower the HVL value of any sample, the better the radiation shielding efficiency due to the lower thickness necessities.

**Figure 9:**
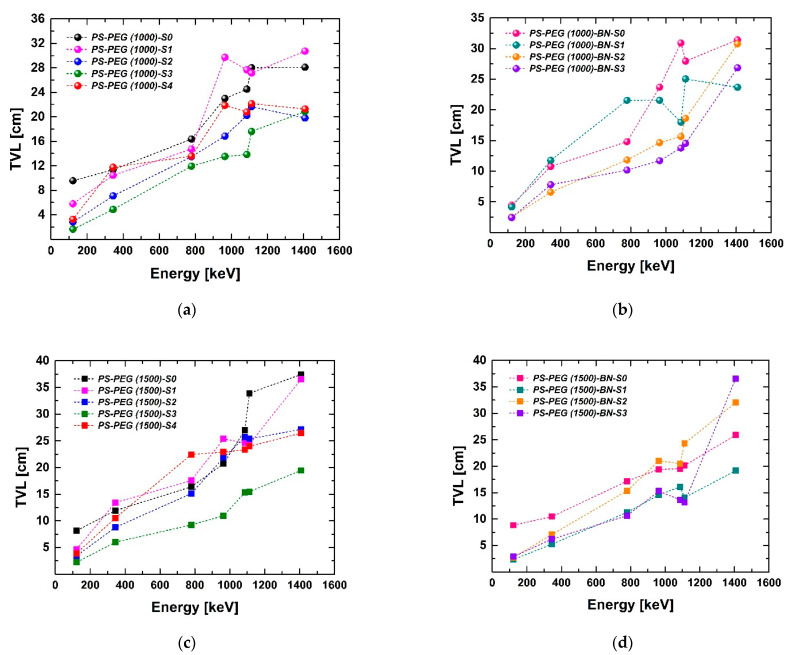

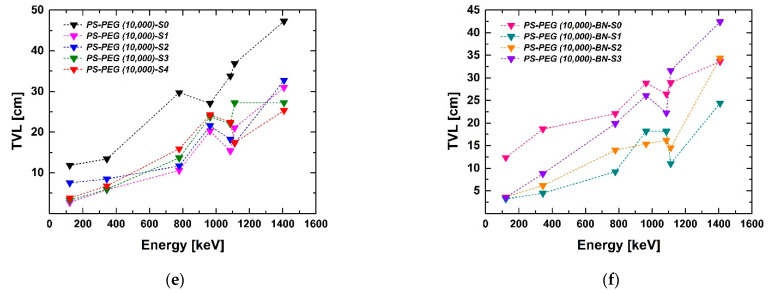
The TVL values of the PbO doped the crosslinked PS-b-PEG block copolymers and the PbO doped the PS-b-PEG-BN nanocomposite materials at different gamma radiation energies (from 121.782 keV to 1408.006 keV) of E 152u: (**a**) PS-PEG (1000)-S0,S1,S2,S3,S4; (**b**) PS-PEG (1000)-BN-S0,S1,S2,S3; (**c**) PS-PEG (1500)-S0,S1,S2,S3,S4; (**d**) PS-PEG (1500)-BN-S0,S1,S2,S3; (**e**) PS-PEG (10,000)-S0,S1,S2,S3,S4; (**f**) PS-PEG (10,000)-BN-S0,S1,S2,S3.

As can be seen in Tables S5 and S6 (tables are available in Supplementary information file; Tables S5 and S6) and Figure 9a–f, the TVL value, such as the HVL value, is a concept that focuses on a similar purpose with a fundamentally different scaling. The tenth value layer is the mean quantity of substance required to absorb 90% of the available radiation, that is, to diminish it to one-tenth of its density. As can be seen in Figure 9, the TVL value, which is the shielding thickness value required to stop 90% of the emitted photons, that is to absorb them, increases as the photon energy increased. These results are a very important marker of the radiation shielding characteristic of the material concerned, i.e., it can be concluded that the lower the TVL value of any sample, the better the radiation shielding efficiency due to the lower thickness necessities. The HVL values of our samples provide persuasive instructions concerning the shielding capacity of our materials in reducing the photon quantities to half of the present situation for the thickness of the samples.

**Figure 10:**
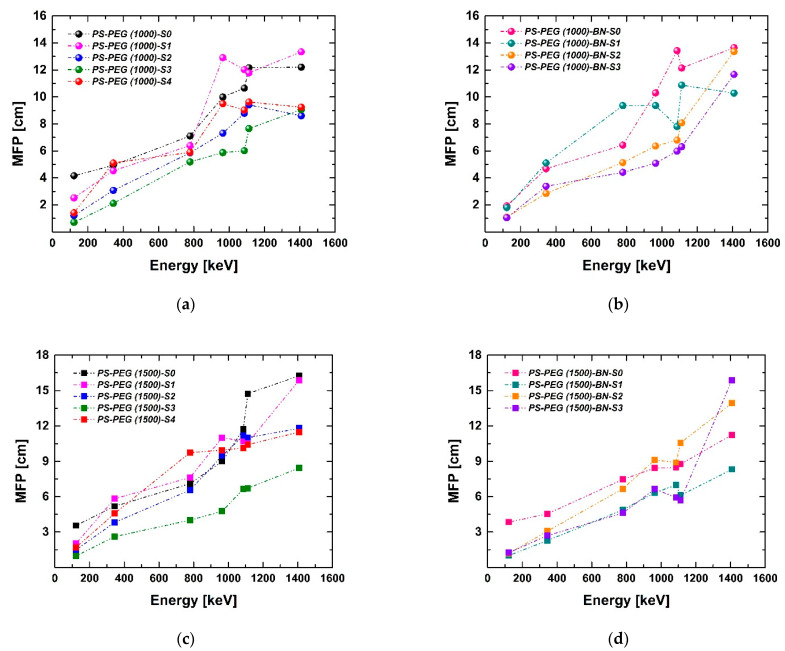

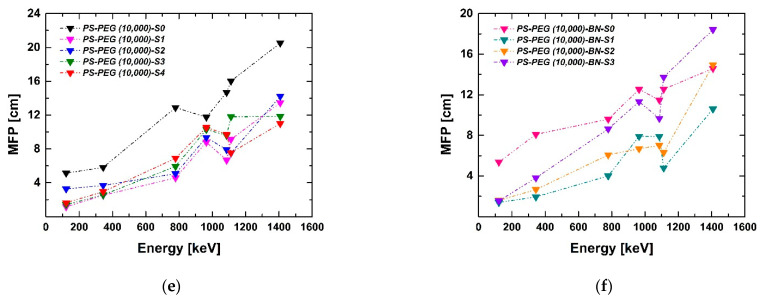
The MFP values of the PbO doped the crosslinked PS-b-PEG block copolymers and the PbO doped the PS-b-PEG-BN nanocomposite materials at different gamma radiation energies (from 121.782 keV to 1408.006 keV) of E 152u: (**a**) PS-PEG (1000)-S0,S1,S2,S3,S4; (**b**) PS-PEG (1000)-BN-S0,S1,S2,S3; (**c**) PS-PEG (1500)-S0,S1,S2,S3,S4; (**d**) PS-PEG (1500)-BN-S0,S1,S2,S3; (**e**) PS-PEG (10,000)-S0,S1,S2,S3,S4; (**f**) PS-PEG (10,000)-BN-S0,S1,S2,S3.

The Mean Free Path (MFP) is the mean span which a photon itinerates in the medium before an interaction happens (or among sequential interactions). As can be seen in Tables S7 and S8 (tables are available in Supplementary information file; Tables S7 and S8) and Figure 10a–f, the acquired values represent that the MFP of materials ascents with photon energy. Additionally, the MFP value is one of the important variables that understandably clarifies the gamma radiation deterioration abilities of the shielding substances used. The smaller the MFP value, the preferable the shielding competence of the substance. Our outcomes demonstrate that the MFP rates of our samples are ascent with photon energy.

**Figure 11:**
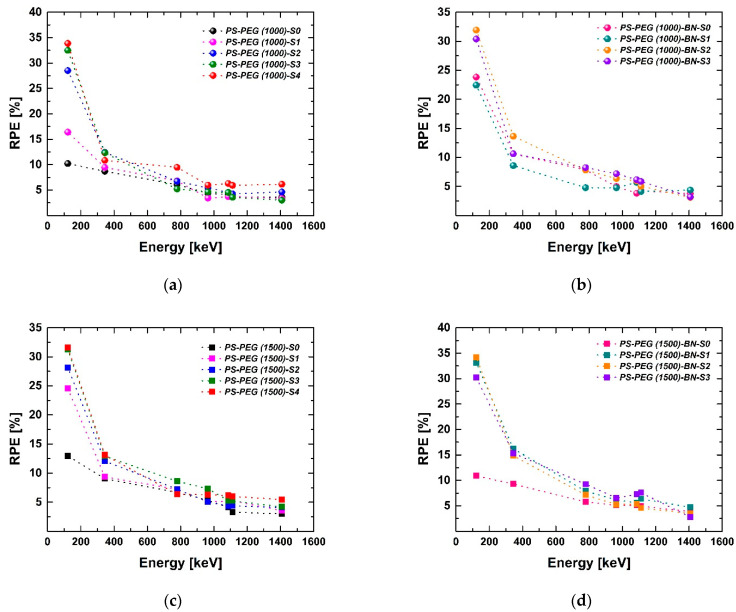

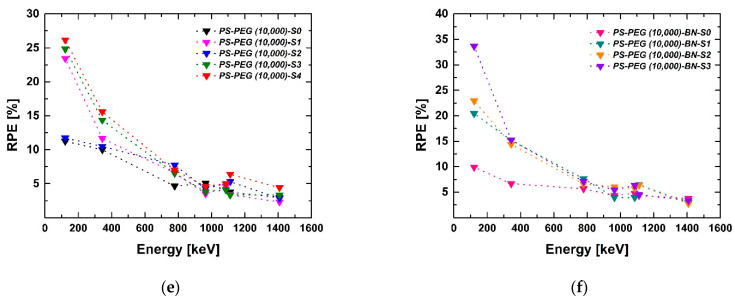
The RPE values of the PbO doped the crosslinked PS-b-PEG block copolymers and the PbO doped the PS-b-PEG-BN nanocomposite materials at different gamma radiation energies (from 121.782 keV to 1408.006 keV) of E 152u: (**a**) PS-PEG (1000)-S0,S1,S2,S3,S4; (**b**) PS-PEG (1000)-BN-S0,S1,S2,S3; (**c**) PS-PEG (1500)-S0,S1,S2,S3,S4; (**d**) PS-PEG (1500)-BN-S0,S1,S2,S3; (**e**) PS-PEG (10,000)-S0,S1,S2,S3,S4; (**f**) PS-PEG (10,000)-BN-S0,S1,S2,S3.

By computing the Radiation Protection Efficiency (RPE), the accomplishment of the materials prepared to determine the attenuation of the gamma photons in the various energy intervals can be monitored. Therefore, in Figure 11a–f, the RPE values for the PbO doped the crosslinked PS-b-PEG block copolymers, and the PbO doped the PS-b-PEG-BN nanocomposite materials have monitored the densities of the photons as a function of different gamma-ray energy intervals. In our work as can be seen in Tables S7 and S8 (table are available in Supplementary information file; Tables S7 and S8) and Figure 11a–f for all our tested composites, our RPE values tend to decrease with increasing energy. These results indicate that our PbO doped polymer-based composites show good shielding performance against gamma radiation. That is, the current changes in doped ratios are quite effective for reducing the intensity of gamma photons.

As it is clearly emphasized in Tables S5–S8 (tables are available in Supplementary information file; Tables S5–S8) and Figure 8, Figure 9, Figure 10 and Figure 11, it has been observed that the measured and calculated values show changes with the change of the polymer type used to improve the gamma radiation shielding materials. In addition, it was observed that the radiation protection capacities of the samples improved when the PbO or BN percentages of the prepared materials were changed.

When the HVL, TVL, MFP, and RPE results of our all samples are examined with a broad perspective, it can be emphasized that these samples developed in our research reveal important and reliable results to enlighten radiation shielding studies. The lower the HVL and TVL values, the better the samples should have. Table S5 (table is available in Supplementary information file; Table S5) shows that as the 121.782–1408.006 keV energy values increase, HVL and TVL values also increase.

When the order of HVL and TVL values for PS-PEG (1000) copolymer and composites are examined: PS-PEG (1000)-S2<PS-PEG (1000)-S3<PS-PEG (1000)-S4<PS-PEG (1000)-S0<PSPEG (1000)-S1. For PS-PEG (1500) copolymer and composites is examined: PS-PEG (1500)-S3<PS-PEG (1500)-S4<PS-PEG (1500)-S2<PS-PEG (1500)-S1<PS-PEG (1500)-S0. For PS-PEG (10,000) copolymer and composites is examined: PS-PEG (10,000)-S4<PS-PEG (10,000)-S3<PS-PEG (10,000)-S1<PS-PEG (10,000)-S2<PS-PEG (10,000)-S0. For PS-PEG (1000)-BN and its composites containing PbO and BN are examined: PS-PEG (1000)-BN-S1<PS-PEG (1000)-BN-S3<PS-PEG (1000)-BN-S2<PS-PEG (1000)-BN-S0. For PS-PEG (1500)-BN and its composites are examined: PS-PEG (1500)-BN-S1<PS-PEG (1500)-BN-S0<PS-PEG (1500)-BN-S2<PS-PEG (1500)-BN-S3. For PS-PEG (10,000)-BN and its composites are examined: PS-PEG (10,000)-BN-S1<PS-PEG (10,000)-BN-S0<PS-PEG (10,000)-BN-S2<PS-PEG (10,000)-BN-S3.

We aim to use the samples that we prepared as radiation shields. Materials that can be used as shields will be preferred in terms of efficiency if they can protect from high levels of radiation with a low thickness. We used TVL and HVL calculations to examine this situation.

As can be seen in Table S5 (table is available in Supplementary information file; Table S5), PS-PEG (1000)-S0, PS-PEG (1500)-S0, and PS-PEG (10,000)-S0 give good values in HVL and TVL calculations, although all of the samples are polymers. Considering the HVL values, which are calculated as the thickness required to absorb half of the incoming radiation, the best value taken for absorbing radiation of 121.782 keV is 2.457 cm. At the high radiation level of 1408.006 keV, the best value is 8.458 cm. At this point, it is understood that we can create a radiation shield only by preparing polymers and this absorption will be efficient with structures that are quite light compared to heavy elements. When we look at the TVL results, which give the thickness required to absorb 90% of the incident radiation, it is seen that the best values of our polymer samples are 8.162 cm for 121.782 keV and 28.097 cm for 1408.006 keV. These calculations, which also show the conclusion that thickness is significantly important in radiation absorption, led us to support our polymers with other metal oxides.

We prepared composites in different proportions by mixing our polymers with lead oxide. When these composites were examined, we found the best value for the HVL value at 121.782 keV radiation with 0.490 cm in our PS-PEG (1000)-S3 sample, which contains 90% lead, as expected. The best HVL value we calculated for 1408.006 keV was 5.856 cm in our PS-PEG (1500)-S3 sample, which also contains 90% lead oxide. When we calculated the TVL value, we obtained our best value in 121.782 keV radiation from our PS-PEG (1000)-S3 sample with 1.629 cm. Our best value for 1408.006 keV was obtained from our PS-PEG (1500)-S3 sample with 19.451 cm. At this point, the contribution of PbO in radiation absorption is seen and it is concluded that a good absorber is obtained when we combine it with our polymer and make it a composite. In addition, being light and flexible, which is one of the characteristics of polymers, highlights the superiority in wrapping a compound such as PbO. On the other hand, when we look at the other results in Table S5 (table is available in Supplementary information file; Table S5), it should be seen that we have created a scale to choose which compound we will use for the shield. In a structure where the weight comes to the fore, a composite with the dense polymer can be preferred, and in a structure where thickness is prominent, a composite with PbO density can be preferred.

On the other hand, the HVL and TVL results of our BN doped samples are given in Table S6 (table is available in Supplementary information file; Table S6). PS-PEG (1000)-BN-S0, PS-PEG (1500)-BN-S0, and PS-PEG (10,000)-BN-S0 samples in the table contain only polymer and BN. When we look at the HVL and TVL values of these samples, we can see that the best HVL value for 121.782 keV is from the PS-PEG (1000)-BN-S0 sample with 1.336 cm and the TVL value with 4.439 cm. It is seen that the best HVL value for 1408.006 keV is 7.801 cm and the best TVL value is 25.913 cm from PS-PEG (1500)-BN-S0 sample. When we compare these values with the values mentioned above, which are only polymers in their structure, the contribution of adding BN to our composite is seen. In addition, BN is thought to be a good neutron absorber. Based on this idea, we can easily conclude that the contribution of BN is important since we think that this mineral we added will also be useful for neutron radiation.

Tables S9 and S10 represent the contrastive outcomes of the successfulness of shielding characteristics of lead oxide and boron-doped materials (tables are available in Supplementary information file; Tables S9 and S10) [28,29,30,31,32,33,34,35,36,37,38,39,40,41,42,43,44,45,46,47,48,49,50,51,52,53,54,55,56,57].

## 4. Conclusions and Future Perspectives

This study includes:

- The polystyrene-co-polyethyleneglycol (PS-PEG) crosslinked block copolymers were constructed from styrene copolymerization by either polyethyleneglycol (PEG) macro crosslinker by using emulsion polymerization under vacuum in the Schlenk system. $M_{\omega }$ values of polyethyleneglycol of PEG were 1000 and 1500. The characterization of the block copolymer was made with the NMR, IR, TGA, and SEM methods. The IR (cm^−1^), 1723.03 (C=O), 1560.04 (C=C), 1103.76 (C–O–C), NMR (δ, ppm); 2.0 (CH_3_), 3.5 (–CH_2_–O–CH_2_), and 5.6-6.2 (vinylic protons). The IR peaks as cm^−1^ in the FTIR spectrum of the crosslinked PS-b-PEG block copolymers are followed: methylene group CH stretching vibrations at 2921.47, benzene ring C=C stretching vibrations at 1600.79, benzene ring overtone band combinations at 1600.79 and 1452.12, –C (C=O) stretching vibrations at 1280.17, and –OCC groups asymmetric stretching vibrations at 1103.73.
- The outcomes of TGA thermograms of the PbO doped the crosslinked PS-b-PEG block copolymers and the PbO doped the PS-b-PEG-BN nanocomposite materials, it is seen that the implemented radiation does not influence the structure of the investigated polymer-based composites. From all TGA results; it can be concluded that PbO increases the durability of the copolymer and BN increases the thermal durability of the copolymer.
- The HVL values of our samples provide persuasive instructions regarding the shielding capacity of our materials, declining the photon amount to half of the present status concerning the thickness of the samples. The acquired values represent that the MFP of materials ascents with photon energy. Additionally, the MFP value is one of the substantial variables that understandably clarifies the gamma radiation deterioration abilities of the shielding substances used. The smaller the MFP rates, the preferable the shielding competence of the material. Our outcomes demonstrate that the MFP rates of our samples are ascent with photon energy. It has been observed that the measured and calculated values show changes with the change of the polymer type used to improve the gamma radiation shielding materials. In addition, it was observed that the radiation protection capacities of the samples improved when the PbO or BN percentages of the prepared materials were changed. When the HVL, TVL, MFP, and RPE results of our all samples are examined with a broad perspective, it can be emphasized that these samples developed in our research reveal important and reliable results to enlighten radiation shielding studies. We observed in our results that the lower the HVL, TVL, and MFP values of any sample, the better the radiation shielding efficiency due to the lower thickness necessities, and the higher the RPE values of any sample, the better the radiation shielding efficiency, i.e., we concluded from our results that the lower the HVL, TVL, and MFP values of any sample, the better the RPE due to the lower thickness necessities.
- It has been observed that the measured and calculated values show changes with the change of the polymer type used to improve the gamma radiation shielding materials. In addition, it was observed that the radiation protection capacities of the samples improved when the PbO or BN percentages of the prepared materials were changed. When we compare all samples, we can deduce that the bigger LACs and MACs rates and ideal absorption of gamma photons are owing to the high percentage rates of PbO ingredients in the substances. If the LACs and MACs results of our all samples are examined with a broad perspective, we can emphasize that these samples developed in our research reveal important and reliable results to enlighten radiation shielding studies. Furthermore, BN was added to our nanocomposites because the neutron absorption property of BN is known. It is expected that this important point will be investigated in further studies and its neutron absorber property will be observed.

It is essential to improve unresearched polymer-based substances with radiation shielding efficiencies. The use of nanocomposite polymeric-based materials for ionizing radiation shielding implementations calls to be investigated. Exhaustive experimental research and trying of such composite materials are crucial before mercantile utilization of the same for radiation shielding implementations. As a result, our research materials reveal that the PbO doped the crosslinked PS-b-PEG block copolymers and the PbO doped the PS-b-PEG-BN nanocomposite materials are fine options for radiation protection objectives for gamma rays that are especially benefitted as a shielding substance for the transportation of radiation sources and an insulation substance for the radioactive waste administration facilities or the building industry. The joining of alternative types of contributions (cement, polymer, metal oxide, etc.) is tremendously crucial in improving the hardness, durability, and radiation absorption capacities of the shielding materials. Polymer structures are a substantial class of substances utilized in radiation shielding investigations since these structures are inexpensive and lightweight, and also it will be the origin of many types of research using composites acquired by suffixing micro or nano oxide, etc., to examine the radiation attenuation theoretically and experimentally.

## Figures and Tables

**Table 1:** Sample ID and compositions (by weight %) of the investigated samples.

Sample ID	PS-b-PEG (wt%)	BN (wt%)	PbO (wt%)
PS-PEG (1000)-S0	100	0	0
PS-PEG (1000)-S1	50	0	50
PS-PEG (1000)-S2	30	0	70
PS-PEG (1000)-S3	10	0	90
PS-PEG (1000)-S4	46.2	0	53.8
PS-PEG (1500)-S0	100	0	0
PS-PEG (1500)-S1	50	0	50
PS-PEG (1500)-S2	30	0	70
PS-PEG (1500)-S3	10	0	90
PS-PEG (1500)-S4	46.2	0	53.8
PS-PEG (10,000)-S0	100	0	0
PS-PEG (10,000)-S1	50	0	50
PS-PEG (10,000)-S2	30	0	70
PS-PEG (10,000)-S3	10	0	90
PS-PEG (10,000)-S4	46.2	0	53.8
PS-PEG (1000)-BN-S0	50	50	0
PS-PEG (1000)-BN-S1	15	15	70
PS-PEG (1000)-BN-S2	5	5	90
PS-PEG(1000)-BN-S3	26.1	13	60.9
PS-PEG (1500)-BN-S0	50	50	0
PS-PEG (1500)-BN-S1	15	15	70
PS-PEG (1500)-BN-S2	5	5	90
PS-PEG (1500)-BN-S3	26.1	13	60.9
PS-PEG (10,000)-BN-S0	50	50	0
PS-PEG (10,000)-BN-S1	15	15	70
PS-PEG (10,000)-BN-S2	5	5	90
PS-PEG (10,000)-BN-S3	26.1	13	60.9

**Table 2:** TGA thermogram values of the PbO doped the crosslinked PS-b-PEG block copolymer and the PS-b-PEG-BN nanocomposite materials.

**PS-PEG (1000)-S0**		**PS-PEG (1000)-S3**		**PS-PEG (1000)-BN-S0**		**PS-PEG (1000)-BN-S2**	
**Temperature (°C)**	**wt (%)**	**Temperature (°C)**	**wt (%)**	**Temperature (°C)**	**wt (%)**	**Temperature (°C)**	**wt (%)**
46.7	76.740	43.3	98.710	46.5	98.800	42.7	96.994
91.0	65.360	85.9	88.730	254.8	96.400	232.8	91.937
254.2	62.480	142.9	85.960	366.9	63.400	352.8	70.028
343.5	49.340	241.8	83.670	424.8	60.00	374.9	64.570
381.2	36.380	279.6	73.250	-	-	-	-
-	-	320.7	66.140	-	-	-	-
-	-	371.3	56.070	-	-	-	-
-	-	402.9	51.820	-	-	-	-
**PS-PEG (1500)-S0**		**PS-PEG (1500)-S3**		**PS-PEG (1500)-BN-S0**		**PS-PEG (1500)-BN-S2**	
**Temperature (** **°C)**	**wt (%)**	**Temperature (** **°C)**	**wt (%)**	**Temperature (** **°C)**	**wt (%)**	**Temperature (** **°C)**	**wt (%)**
53.4	93.500	47.2	98.070	37.0	99.000	53.8	99.000
322.4	82.500	78.4	88.290	281.9	95.300	88.8	97.800
417.9	-0.500	165.9	85.930	379.6	63.400	152.9	97.000
-	-	244.4	83.250	418.7	61.200	238.7	94.100
-	-	275.5	74.360	-	-	322.9	76.700
-	-	349.0	61.040	-	-	357.9	72.000
-	-	370.1	53.890	-	-	421.0	69.400
**PS-PEG (10,000)-S0**		**PS-PEG (10,000)-S3**		**PS-PEG (10,000)-BN-S0**		**PS-PEG (10,000)-BN-S2**	
**Temperature (** **°C)**	**wt (%)**	**Temperature (** **°C)**	**wt (%)**	**Temperature (** **°C)**	**wt (%)**	**Temperature (** **°C)**	**wt (%)**
31.2	96.060	44.0	97.660	51.8	99.200	65.8	99.00
66.5	76.020	72.0	90.730	272.8	96.600	85.1	97.600
293.8	69.250	83.9	89.500	365.3	75.900	236.7	95.100
401.6	3.000	231.9	87.200	417.3	74.800	336.1	77.100
-	-	282.7	77.160	-	-	377.2	68.800
-	-	372.0	56.240	-	-	412.4	68.800
-	-	-	-	-	-	-	-

## Data Availability

The data presented in this study are available on request from the corresponding author.

## Supplementary Materials

The following are available online at https://www.mdpi.com/article/10.3390/polym13193246/s1, Section S1: Basic Information of Gamma-Irradiation Theory, Figure S1: detector schema for gamma irradiation attenuation fruitfulness evaluations, Figure S2: SEM photographs of PS-b-PEG (1000) block copolymer (100% PS-PEG (1000)+0% BN+0% PbO in Table 1): (a) PS-PEG (1000)-S0 (4900 × magnification); (b) PS-PEG (1000)-S0 (10,210 × magnification), Figure S3: SEM photographs of the PS-b-PEG (1500) block copolymer (100% PS-PEG (1500)+0% BN+0% PbO in Table 1): (a) PS-PEG (1500)-S0 (10,000 × magnification); (b) PS-PEG (1500)-S0 (1370 × magnification), Figure S4: SEM photographs of the crosslinked PS-b-PEG (10,000) block copolymer (100% PS-PEG (10,000)+0% BN+0% PbO in Table 1): (a) PS-PEG (10,000)-S0 (5000 × magnification); (b) PS-PEG (10,000)-S0 (1000 × magnification), Figure S5: SEM photographs of the crosslinked PS-b-PEG (1000) block copolymer+BN nanocomposite (50% PS-PEG (1000)+50% BN+0% PbO in Table 1): (a) PS-PEG (1000)-BN-S0 (5000 × magnification); (b) PS-PEG (1000)-BN-S0 (1750 × magnification), Figure S6: SEM photographs of the crosslinked PS-b-PEG (1500) block copolymer+BN nanocomposite (50% PS-PEG (1500)+50% BN+0% PbO in Table 1): (a) PS-PEG (1500)-BN-S0 (4000 × magnification); (b) PS-PEG (1500)-BN-S0 (4000 × magnification), Figure S7: SEM photographs of the crosslinked PS-b-PEG (10,000) block copolymer+BN nanocomposite (50% PS-PEG (10,000)+50% BN+0% PbO in Table 1): (a) PS-PEG (10,000)-BN-S0 (12,000 × magnification); (b) PS-PEG (10,000)-BN-S0 (4480 × magnification), Figure S8: SEM photographs of the crosslinked PS-b-PEG (1000) block copolymer+PbO nanocomposite (10% PS-PEG (1000)+0% BN+90% PbO in Table 1): (a) PS-PEG (1000)-S3 (11,980 × magnification); (b) PS-PEG (1000)-S3 (3950 × magnification), Figure S9: SEM photographs of the crosslinked PS-b-PEG (1000) block copolymer+BN+PbO nanocomposite (5% PS-PEG (1000)+5% BN+90% PbO in Table 1): (a) PS-PEG (1000)-BN-S2 (12,000 × magnification); (b) PS-PEG (1000)-BN-S2 (15,000 × magnification), Figure S10: SEM photographs of the crosslinked PS-b-PEG (1500) block copolymer+PbO nanocomposite (10% PS-PEG (1500)+0% BN+90% PbO): (a) PS-PEG (1500)-S3 (15800 × magnification); (b) PS-PEG (1500)-S3 (10,710 × magnification), Figure S11: SEM photographs of the crosslinked PS-b-PEG (1500) block copolymer+BN+PbO nanocomposite (5% PS-PEG (1500)+5% BN+90% PbO in Table 1): (a) PS-PEG (1500)-BN-S2 (1750 × magnification); (b) PS-PEG (1500)-BN-S2 (3000 × magnification), Figure S12: SEM photographs of the crosslinked PS-b-PEG (10,000) block copolymer+PbO nanocomposite (10% PS-PEG (10,000)+0% BN+90% PbO in Table 1): (a) PS-PEG (10,000)-S3 (5000 × magnification); (b) PS-PEG (10,000)-S3 (7500 × magnification), Figure S13: SEM photographs of the crosslinked PS-b-PEG (10,000) block copolymer+BN+PbO nanocomposite (5% PS-PEG (10,000)+5% BN+90% PbO in Table 1): (a) PS-PEG (10,000)-BN-S2 (15,000 × magnification); (b) PS-PEG (10,000)-BN-S2 (1500 × magnification), Table S1: the linear attenuation coefficients (LACs) of the PbO doped the crosslinked PS-b-PEG block copolymers samples, Table S2: the linear attenuation coefficients (LACs) of the PbO doped the PS-b-PEG-BN nanocomposite samples, Table S3: the mass attenuation coefficients (MACs) of the PbO doped the crosslinked PS-b-PEG block copolymers samples, Table S4: the mass attenuation coefficients (MACs) of the PbO doped the crosslinked PS-b-PEG-BN block copolymers samples, Table S5: the half value layer (HVL) and the tenth value layer (TVL) values of the PbO doped the crosslinked PS-b-PEG block copolymers samples, Table S6: the half value layer (HVL) and the tenth value layer (TVL) values of the PbO doped the crosslinked PS-b-PEG-BN block copolymers samples, Table S7: the mean free path (MFP) and the radiation protection efficiency (RPE) values of the PbO doped the crosslinked PS-b-PEG block copolymers samples, Table S8: the mean free path (MFP) and the radiation protection efficiency (RPE) values of the PbO doped the crosslinked PS-b-PEG-BN block copolymers samples, Table S9: comparative informations of PbO doped radiation shielding characteristics, Table S10: comparative informations of boron-doped radiation shielding characteristics.
