# Supplementary material for: Gamma Irradiation and the Radiation Shielding Characteristics: For the Lead Oxide Doped the Crosslinked Polystyrene-b-Polyethyleneglycol Block Copolymers and the Polystyrene-b-Polyethyleneglycol-Boron Nitride Nanocomposites
Source: Polymers (Basel). 2021 Sep 24;13(19):3246. doi: 10.3390/polym13193246 (PMC8512662; doi:10.3390/polym13193246)
Supplement: Supplementary file 1 [file polymers-13-03246-s001.zip › polymers-1397322-supplementary.pdf]

polymers

SUPPLEMENTARY INFORMATION FILE

S1. Basic Information of Gamma-Irradiation Theory

A photon with beginning intensity I_0 falls to its attenuated I intensity after exceeding from the sample. This reduction is elucidated via Lambert-Beer's law [43]:

$$\mu = -[\ln{(I/I_0)}/x] \tag{S1}$$

here μ characterize the attenuation coefficient of the material and x is the thickness of the absorber. After designating experimental μ values, mass attenuation coefficients μ/ρ (MACs) of the analyzed materials are computed by dividing the μ with the intensity (μ/ρ) of the materials [44]:

$$\mu_m = \frac{\mu}{\rho} = \sum w_i \left(\frac{\mu}{\rho}\right)_i \tag{S2}$$

here w_i and $(\mu/\rho)_i$ elucidate the weight component and MACs for each element in the material. Many important terms such as the total atomic and electronic cross-sections, effective atomic number, and electron density can be calculated by this expression.

The total atomic cross-section (σ_a)(cm^2/atom) can be elucidated as follows [45]:

$$\sigma_a = \frac{(\mu_m)}{N_A \sum_i w_i/A_i} \tag{S3}$$

The total electronic cross-section (σ_e)(cm^2/electron) can be elucidated as follows [45]:

$$\sigma_e = \frac{1}{N}\sum_i \mu_{mi}\frac{f_i A_i}{Z_i} \tag{S4}$$

here Z_i is the atomic number and f_i is the abundance ratio of the element.

Effective atomic number (Z_{eff}) of the composites can be ejected from σ_a and σ_e via the following expression [45]:

$$Z_{eff} = \frac{\sigma_a}{\sigma_e} \tag{S5}$$

Electron density (N_E) is elucidated as the number of electrons per unit mass of the reacting matter [45]:

$$N_E = (n_{tot}N)\frac{Z_{eff}}{A_{tot}} \tag{S6}$$

The half-value layer (HVL) and tenth value layer (TVL) thickness ensure crucial knowledge concerning radiation protection as well as the influence intensity of radiation at alternative gamma energy regions implemented to the developed composites. The HVL is characterized as the beginning intensity of the same kind of absorber that decreases the restricted gamma-ray intensity to one-half of the main intensity and this layer thickness (cm) is elucidated as follows [46-47]:

$$HVL = X_h = \frac{\ln 2}{\mu} \approx \frac{0.693}{\mu} \tag{S7}$$

The TVL is characterized as the beginning intensity of the same kind of absorber that decreases the restricted gamma-ray intensity to one-tenth of the main intensity and this layer thickness (cm) is elucidated as follows [47]:

$$TVL = X_t = \frac{\ln 10}{\mu} \approx \frac{2.303}{\mu} \tag{S8}$$

The Mean Free Path (MFP) value of a photon is the mean interval energy of a photon that itinerates along with a dedicated absorber before incurring an interaction and this

parameter has a crucial impact on the exponential calculation of the gamma photon and this expression can be elucidated as follows [48]:

$$MFP = X_m = \frac{1}{\mu} \tag{S9}$$

Furthermore, when we request to investigate the radiation protection efficiency (RPE) of whichever materials, we can elucidate this value as following [48]:

$$RPE(\%) = \left(1 - \frac{I}{I_0}\right) \times 100 \tag{S10}$$

Experimental uncertainties in the MACs computations can be conjecture as follow: [44,48]:

$$\Delta\mu_m = \frac{1}{\rho x}\sqrt{\left(\frac{\Delta I}{I}\right)^2 + \left(\frac{\Delta I_0}{I_0}\right)^2 + \ln\left(\frac{\Delta I}{I}\right)^2\left(\frac{\Delta I_0}{I_0}\right)^2} \tag{S11}$$

here ρ is the density of the sample, ΔI_0 and ΔI is the indefiniteness for initial (I_0) and attenuated (I) countings.

Assorted programs are existing to compute the MACs to whichever material by substituting its chemical formula or weight fraction of its constituent elements for the broad spacing of energy 1keV to 100GeV. The XCOM computer program was cultivated by Berger and Hubbell [49] to compute the MACs and cross-section of elements, compounds, and mixtures for dissimilar photon energies (1 keV to 100GeV). Additionally, the XCOM from DOS converted to Windows operating system; the design of the windows named WinXCom was developed by Gerward et al. [50,51]. WinXCom program was benefited from this work theoretically to compute μm by substituting the necessary weight component.

S2. Gamma Ray Attenuation Measurements

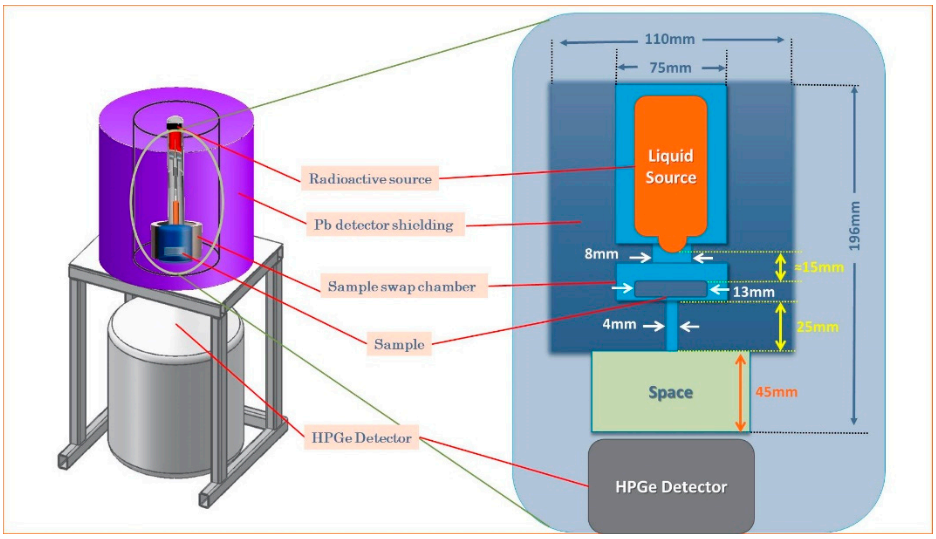

Figure S1. Detector schema for gamma irradiation attenuation fruitfulness evaluations.

S3. Morphological Characterization of the PbO Doped the Crosslinked PS-b-PEG Block Copolymers and the PbO Doped the PS-b-PEG Nanocomposite Materials

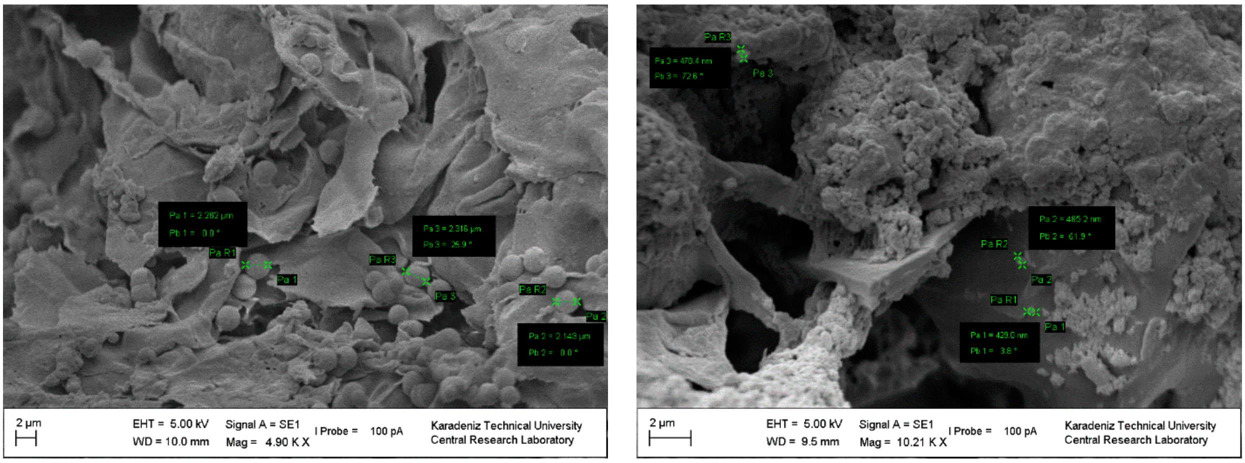

(**a**) (**b**)

Figure S2. SEM photographs of PS-b-PEG (1000) block copolymer (100% PS-PEG (1000)+0% BN+0% PbO in Table 1): (**a**) PS-PEG (1000)-S0 (4900 × magnification); (**b**) PS-PEG (1000)-S0 (10,210 × magnification).

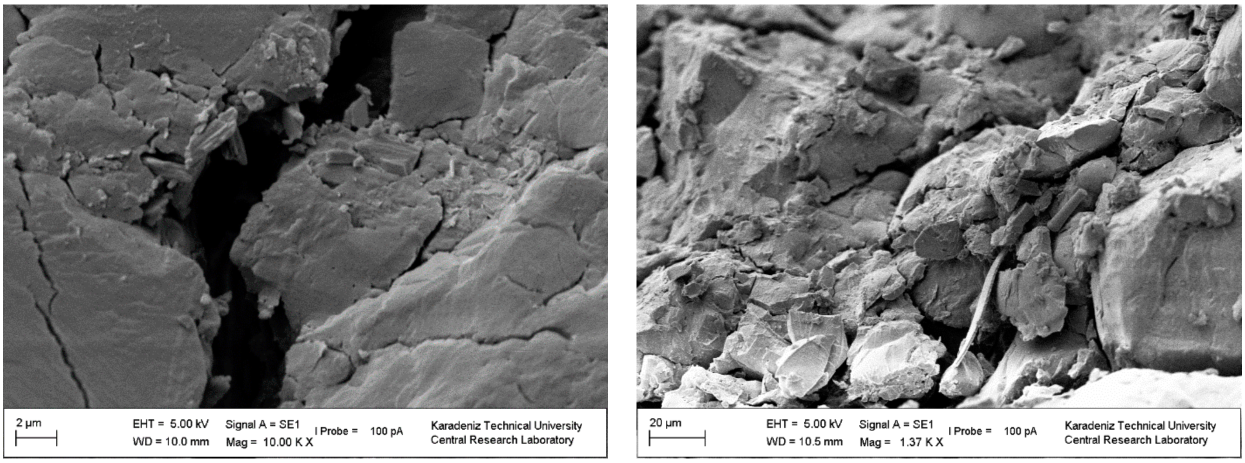

(**a**) (**b**)

Figure S3. SEM photographs of the PS-b-PEG (1500) block copolymer (100% PS-PEG (1500)+0% BN+0% PbO in Table 1): (**a**) PS-PEG (1500)-S0 (10,000 × magnification); (**b**) PS-PEG (1500)-S0 (1370 × magnification).

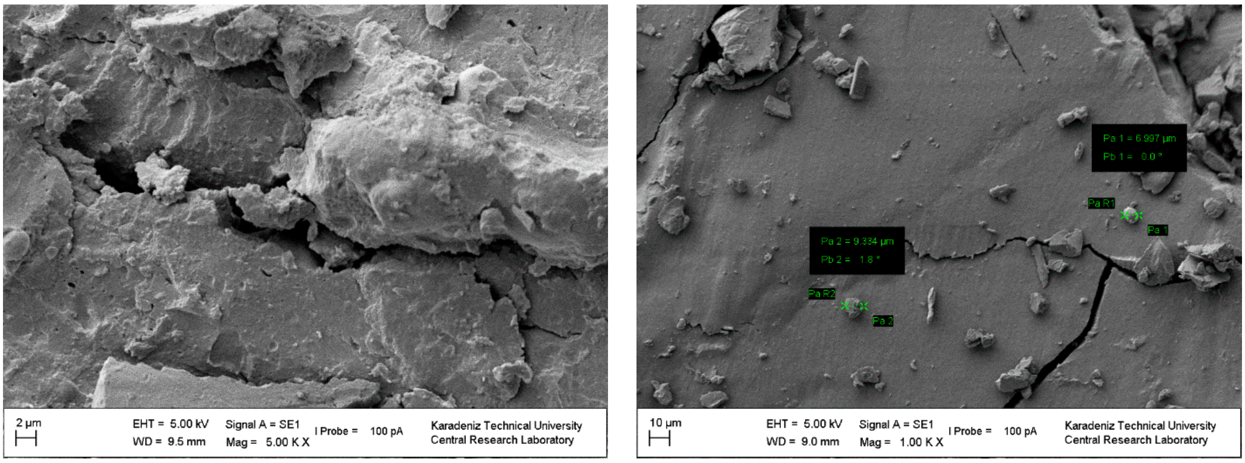

(**a**) (**b**)

Figure S4. SEM photographs of the crosslinked PS-b-PEG (10,000) block copolymer (100% PS-PEG (10,000)+0% BN+0% PbO in Table 1): (**a**) PS-PEG (10,000)-S0 (5000X magnification); (**b**) PS-PEG (10,000)-S0 (1000X magnification).

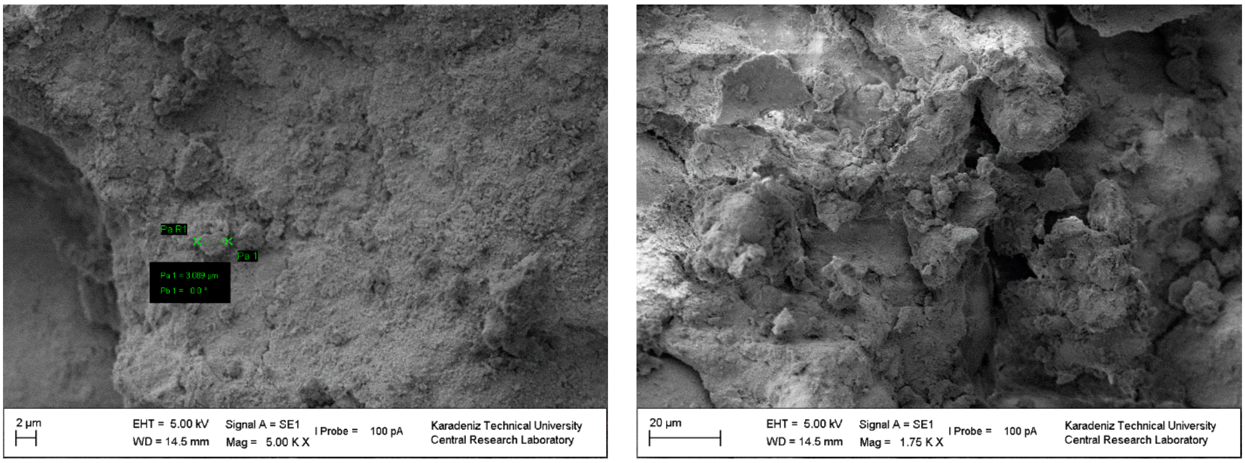

(**a**) (**b**)

Figure S5. SEM photographs of the crosslinked PS-b-PEG (1000) block copolymer+BN nanocomposite (50% PS-PEG (1000)+50% BN+0% PbO in Table 1): (**a**) PS-PEG (1000)-BN-S0 (5000 × magnification); (**b**) PS-PEG (1000)-BN-S0 (1750 × magnification).

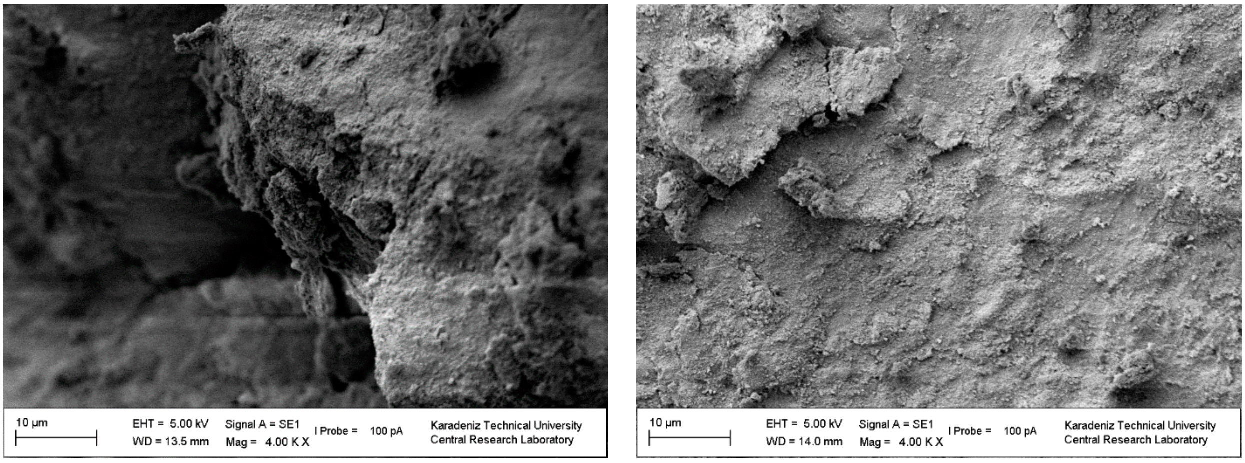

(**a**) (**b**)

Figure S6. SEM photographs of the crosslinked PS-b-PEG (1500) block copolymer+BN nanocomposite (50% PS-PEG (1500)+50% BN+0% PbO in Table 1): (**a**) PS-PEG (1500)-BN-S0 (4000 × magnification); (**b**) PS-PEG (1500)-BN-S0 (4000 × magnification).

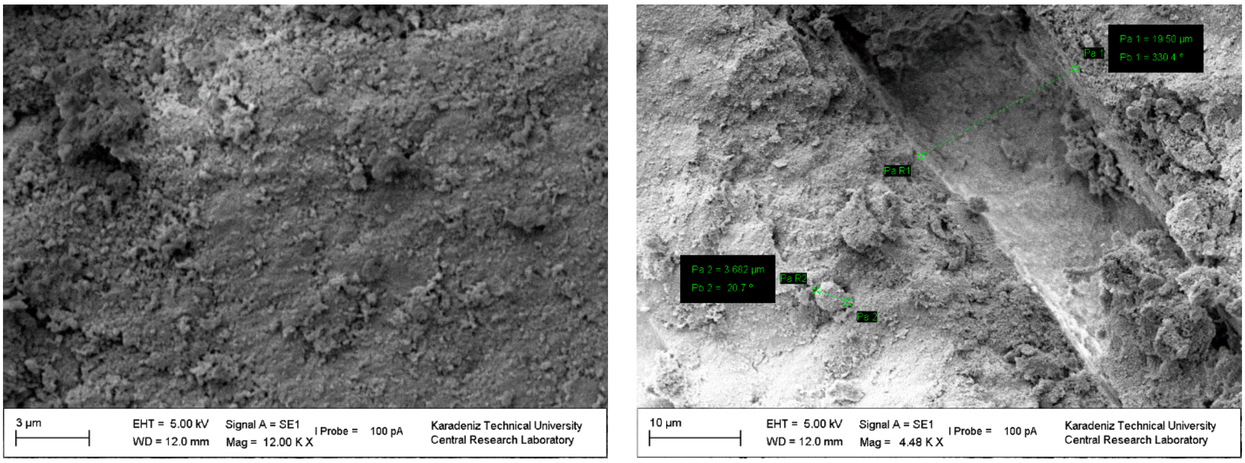

(**a**) (**b**)

Figure S7. SEM photographs of the crosslinked PS-b-PEG (10,000) block copolymer+BN nanocomposite (50% PS-PEG (10,000)+50% BN+0% PbO in Table 1): (**a**) PS-PEG (10,000)-BN-S0 (12,000 × magnification); (**b**) PS-PEG (10,000)-BN-S0 (4480 × magnification).

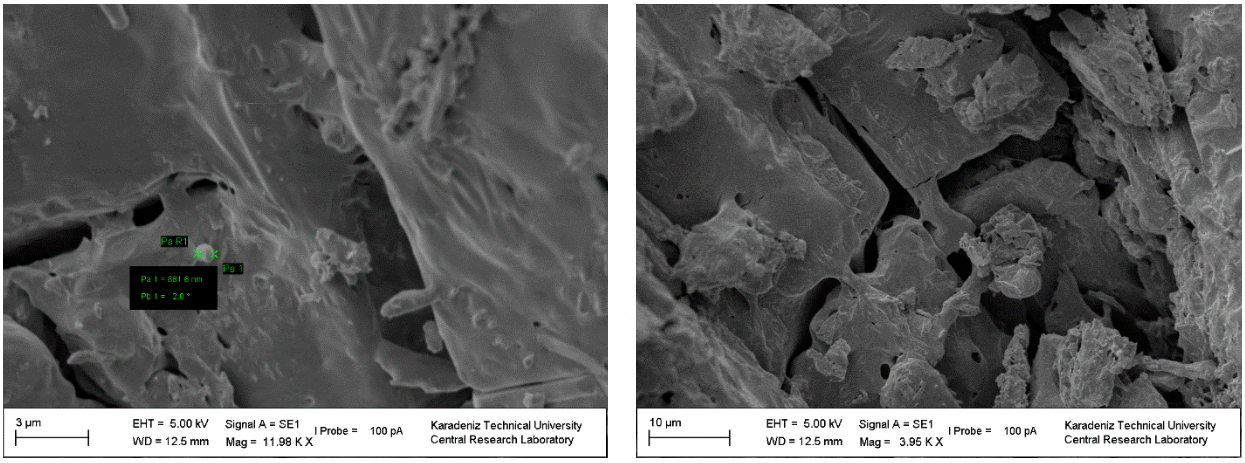

(**a**) (**b**)

Figure S8. SEM photographs of the crosslinked PS-b-PEG (1000) block copolymer+PbO nanocomposite (10% PS-PEG (1000)+0% BN+90% PbO in Table 1): (**a**) PS-PEG (1000)-S3 (11,980 × magnification); (**b**) PS-PEG (1000)-S3 (3950 × magnification).

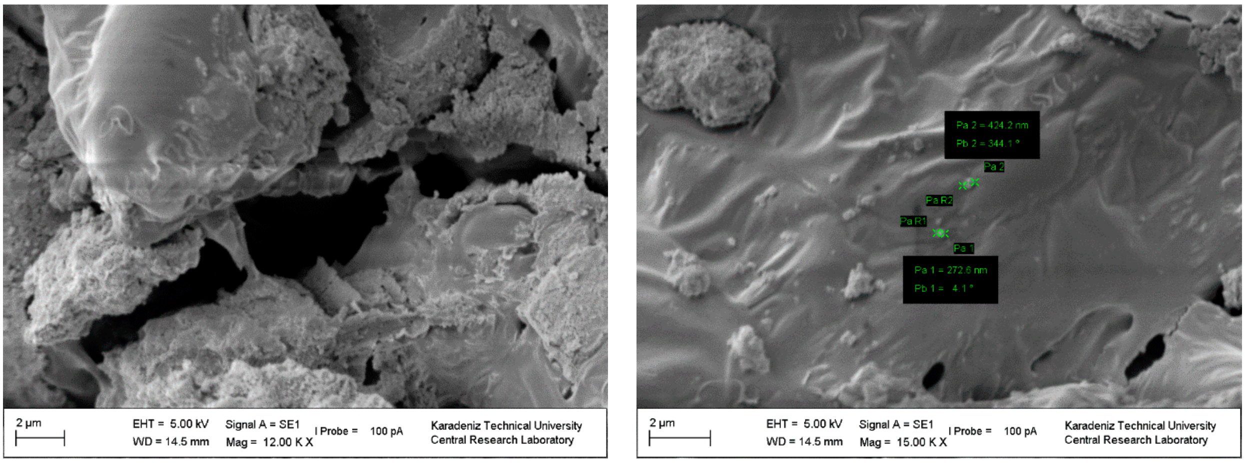

(**a**) (**b**)

Figure S9. SEM photographs of the crosslinked PS-b-PEG (1000) block copolymer+BN+PbO nanocomposite (5% PS-PEG (1000)+5% BN+90% PbO in Table 1): (**a**) PS-PEG (1000)-BN-S2 (12,000 × magnification); (**b**) PS-PEG (1000)-BN-S2 (15,000 × magnification).

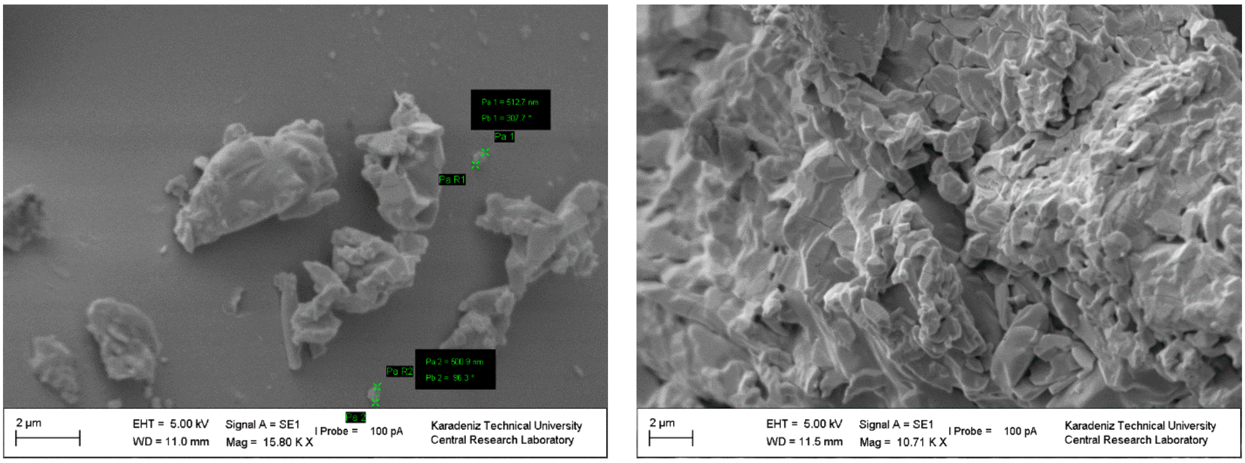

(**a**) (**b**)

Figure S10. SEM photographs of the crosslinked PS-b-PEG (1500) block copolymer+PbO nanocomposite (10% PS-PEG (1500)+0% BN+90% PbO): (**a**) PS-PEG (1500)-S3 (15,800 × magnification); (**b**) PS-PEG (1500)-S3 (10,710 × magnification).

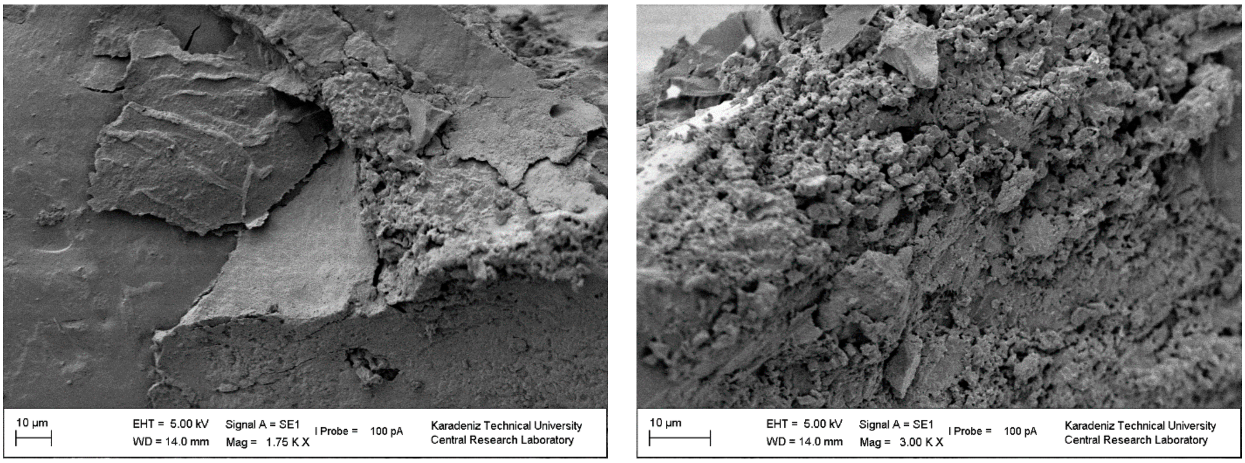

(**a**) (**b**)

Figure S11. SEM photographs of the crosslinked PS-b-PEG (1500) block copolymer+BN+PbO nanocomposite (5% PS-PEG (1500)+5% BN+90% PbO in Table 1): (**a**) PS-PEG (1500)-BN-S2 (1750 × magnification); (**b**) PS-PEG (1500)-BN-S2 (3000 × magnification).

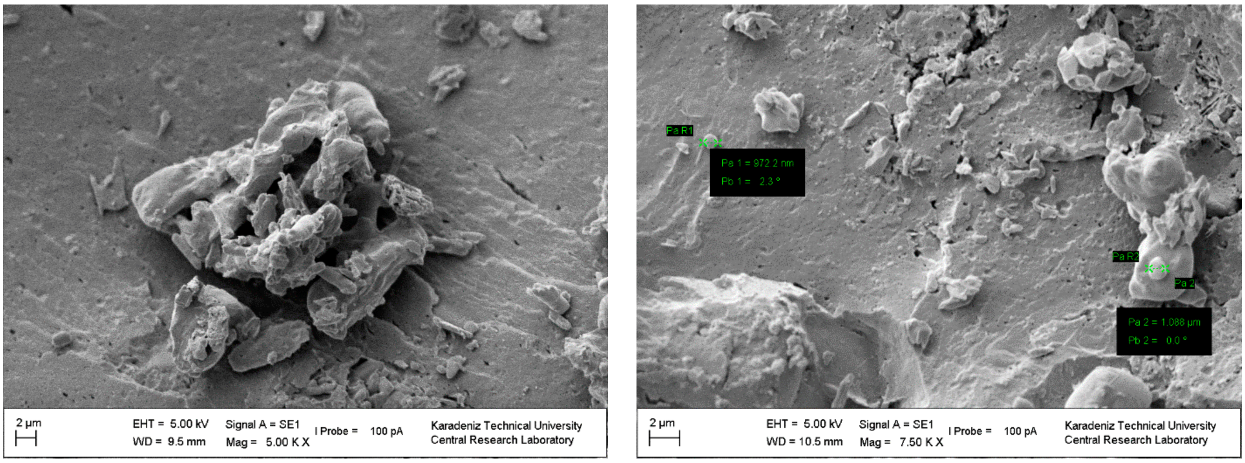

(**a**) (**b**)

Figure S12. SEM photographs of the crosslinked PS-b-PEG (10,000) block copolymer+PbO nanocomposite (10% PS-PEG (10,000)+0% BN+90% PbO in Table 1): (**a**) PS-PEG (10,000)-S3 (5000 × magnification); (**b**) PS-PEG (10,000)-S3 (7500 × magnification).

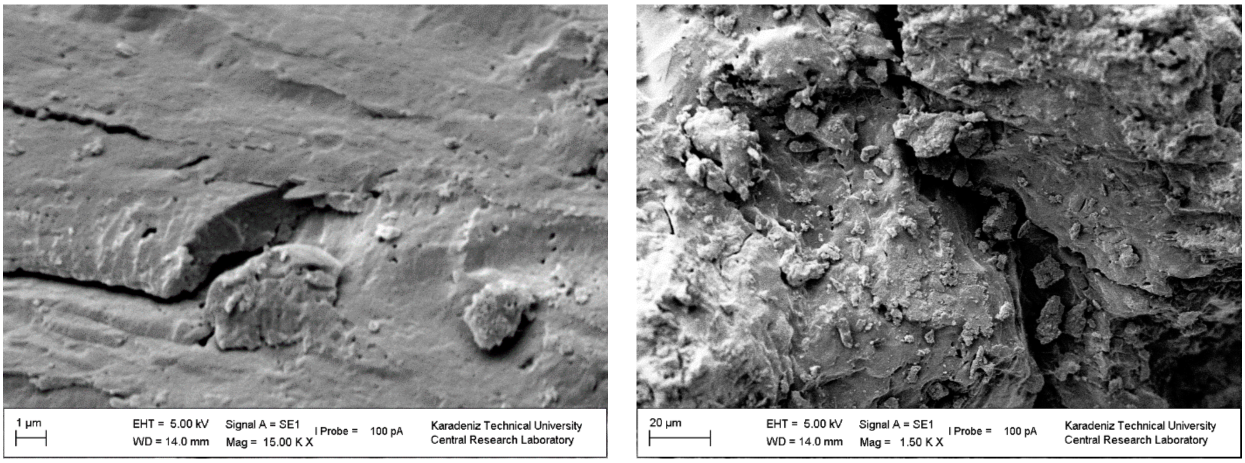

(**a**) (**b**)

Figure S13. SEM photographs of the crosslinked PS-b-PEG (10,000) block copolymer+BN+PbO nanocomposite (5% PS-PEG (10,000)+5% BN+90% PbO in Table 1): (**a**) PS-PEG (10,000)-BN-S2 (15,000 × magnification); (**b**) PS-PEG (10,000)-BN-S2 (1500 × magnification).

S4. Gamma-Ray Attenuation Characteristics of the PbO Doped the Crosslinked PS-b-PEG Block Copolymers and the PbO Doped the PS-b-PEG-BN Nanocomposite Materials

S4.1. Linear (μ_{linear}) and Mass Attenuation (μ_{mass}) Coefficients

Table S1. The linear attenuation coefficients (LACs) of the PbO doped the crosslinked PS-b-PEG block copolymers samples.

**Sample ID**	**PS-PEG (1000)-S0**		**PS-PEG (1000)-S1**		**PS-PEG (1000)-S2**		**PS-PEG (1000)-S3**		**PS-PEG (1000)-S4**	
	$\mu_L\ (cm^{-1})$									
**Photon Energy (keV)**	**XCOM**	**EXP.**	**XCOM**	**EXP.**	**XCOM**	**EXP.**	**XCOM**	**EXP.**	**XCOM**	**EXP.**
121.782	0,273	0.241	2.837	0.397	4.292	0.823	7.957	1.414	3.047	0.704
344.279	0.193	0.203	0.345	0.221	0.453	0.325	0.764	0.473	0.172	0.196
778.904	0.136	0.141	0.145	0.156	0.165	0.171	0.248	0.193	0.333	0.170
964.079	0.123	0.100	0.123	0.077	0.138	0.137	0.203	0.170	0.124	0.105
1085.869	0.116	0.094	0.114	0.083	0.126	0.114	0.184	0.166	0.114	0.111
1112.074	0.115	0.082	0.112	0.085	0.124	0.106	0.180	0.131	0.112	0.104
1408.006	0.101	0.082	0.097	0.075	0.106	0.116	0.152	0.110	0.097	0.108
**Sample ID**	**PS-PEG (1500)-S0**		**PS-PEG (1500)-S1**		**PS-PEG (1500)-S2**		**PS-PEG (1500)-S3**		**PS-PEG (1500)-S4**	
	$\mu_L\ (cm^{-1})$									
**Photon Energy (keV)**	**XCOM**	**EXP.**	**XCOM**	**EXP.**	**XCOM**	**EXP.**	**XCOM**	**EXP.**	**XCOM**	**EXP.**
121.782	0.265	0.282	2.875	0.493	3.659	0.672	5.666	1.039	2.996	0.590
344.279	0.186	0.193	0.350	0.172	0.386	0.262	0.544	0.384	0.169	0.218
778.904	0.132	0.141	0.147	0.131	0.141	0.153	0.177	0.249	0.327	0.103
964.079	0.119	0.111	0.125	0.091	0.118	0.106	0.145	0.210	0.122	0.101
1085.869	0.112	0.085	0.115	0.093	0.107	0.089	0.131	0.151	0.112	0.099

1112.074	0.111	0.068	0.114	0.095	0.105	0.091	0.128	0.149	0.110	0.096
1408.006	0.099	0.062	0.098	0.063	0.090	0.085	0.109	0.118	0.095	0.087
**Sample ID**	**PS-PEG (10,000)-S0**		**PS-PEG(10,000)-S1**		**PS-PEG (10,000)-S2**		**PS-PEG (10,000)-S3**		**PS-PEG (10,000)-S4**	
**Photon Energy (keV)**	$\mu_L\ (cm^{-1})$									
	**XCOM**	**EXP.**	**XCOM**	**EXP.**	**XCOM**	**EXP.**	**XCOM**	**EXP.**	**XCOM**	**EXP.**
121.782	0.200	0.195	4.134	0.853	4.305	0.306	5.564	0.714	3.480	0.609
344.279	0.141	0.172	0.503	0.396	0.454	0.271	0.534	0.387	0.410	0.340
778.904	0.100	0.078	0.211	0.218	0.165	0.197	0.173	0.169	0.167	0.145
964.079	0.088	0.085	0.189	0.114	0.143	0.107	0.143	0.097	0.149	0.095
1085.869	0.085	0.068	0.166	0.150	0.126	0.127	0.128	0.104	0.131	0.103
1112.074	0.084	0.062	0.163	0.110	0.124	0.133	0.126	0.085	0.128	0.132
1408.006	0.074	0.049	0.141	0.074	0.106	0.070	0.107	0.085	0.111	0.091

Table S2. The linear attenuation coefficients (LACs) of the PbO doped the PS-b-PEG-BN nanocomposite samples.

**Sample ID**	**PS-PEG (1000)-BN-S0**		**PS-PEG (1000)-BN-S1**		**PS-PEG (1000)-BN-S2**		**PS-PEG (1000)-BN-S3**	
**Photon Energy (keV)**	$\mu_L\ (cm^{-1})$							
	**XCOM**	**EXP.**	**XCOM**	**EXP.**	**XCOM**	**EXP.**	**XCOM**	**EXP.**
121.782	0.212	0.519	3.736	0.554	5.158	0.920	4.414	0.953
344.279	0.150	0.214	0.391	0.196	0.494	0.351	0.490	0.296
778.904	0.106	0.156	0.141	0.107	0.160	0.195	0.188	0.226
964.079	0.096	0.097	0.118	0.106	0.131	0.157	0.159	0.197
1085.869	0.090	0.075	0.108	0.128	0.118	0.147	0.146	0.167
1112.074	0.089	0.082	0.106	0.092	0.116	0.124	0.143	0.158
1408.006	0.079	0.073	0.090	0.097	0.098	0.075	0.123	0.086
**Sample ID**	**PS-PEG (1500)-BN-S0**		**PS-PEG (1500)-BN-S1**		**PS-PEG (1500)-BN-S2**		**PS-PEG (1500)-BN-S3**	
**Photon Energy (keV)**	$\mu_L\ (cm^{-1})$							
	**XCOM**	**EXP.**	**XCOM**	**EXP.**	**XCOM**	**EXP.**	**XCOM**	**EXP.**
121.782	0.250	0.260	4.355	0.998	4.448	0.846	3.782	0.801
344.279	0.176	0.220	0.456	0.439	0.426	0.326	0.420	0.371
778.904	0.125	0.134	0.165	0.205	0.138	0.150	0.161	0.217
964.079	0.113	0.119	0.138	0.158	0.113	0.110	0.136	0.151
1085.869	0.106	0.118	0.126	0.143	0.102	0.112	0.125	0.169
1112.074	0.105	0.114	0.123	0.164	0.100	0.095	0.123	0.175
1408.006	0.093	0.089	0.106	0.120	0.085	0.072	0.105	0.063
**Sample ID**	**PS-PEG (10,000)-BN-S0**		**PS-PEG (10,000)-BN-S1**		**PS-PEG (10,000)-BN-S2**		**PS-PEG (10,000)-BN-S3**	
**Photon Energy (keV)**	$\mu_L\ (cm^{-1})$							
	**XCOM**	**EXP.**	**XCOM**	**EXP.**	**XCOM**	**EXP.**	**XCOM**	**EXP.**
121.782	0.204	0.186	3.106	0.717	5.379	0.627	2.693	0.648
344.279	0.141	0.123	0.325	0.515	0.515	0.375	0.299	0.262
778.904	0.100	0.104	0.118	0.249	0.167	0.165	0.115	0.116
964.079	0.090	0.080	0.098	0.127	0.137	0.150	0.097	0.088
1085.869	0.085	0.087	0.090	0.127	0.123	0.142	0.089	0.103

1112.074	0.084	0.079	0.088	0.209	0.121	0.158	0.087	0.073
1408.006	0.075	0.069	0.075	0.094	0.102	0.067	0.075	0.054

Table S3. The mass attenuation coefficients (MACs) of the PbO doped the crosslinked PS-b-PEG block copolymers samples.

**Sample ID**	**PS-PEG (1000)-S0**		**PS-PEG (1000)-S1**		**PS-PEG (1000)-S2**		**PS-PEG (1000)-S3**		**PS-PEG (1000)-S4**	
	$\mu_m\ (cm^2/g)$									
**Photon Energy (keV)**	**XCOM**	**EXP.**	**XCOM**	**EXP.**	**XCOM**	**EXP.**	**XCOM**	**EXP.**	**XCOM**	**EXP.**
121.782	0.268	0.235	1.879	0.261	2.345	0.449	3.701	0.656	1.884	0.435
344.279	0.188	0.198	0.229	0.145	0.247	0.177	0.355	0.220	0.106	0.121
778.904	0.133	0.137	0.096	0.103	0.090	0.093	0.115	0.090	0.206	0.105
964.079	0.120	0.098	0.082	0.051	0.075	0.075	0.094	0.079	0.077	0.065
1085.869	0.114	0.092	0.075	0.055	0.069	0.062	0.085	0.077	0.071	0.068
1112.074	0.112	0.080	0.074	0.056	0.068	0.058	0.084	0.061	0.069	0.064
1408.006	0.099	0.080	0.064	0.049	0.059	0.063	0.071	0.051	0.059	0.067
**Sample ID**	**PS-PEG (1500)-S0**		**PS-PEG (1500)-S1**		**PS-PEG (1500)-S2**		**PS-PEG (1500)-S3**		**PS-PEG (1500)-S4**	
	$\mu_m\ (cm^2/g)$									
**Photon Energy (keV)**	**XCOM**	**EXP.**	**XCOM**	**EXP.**	**XCOM**	**EXP.**	**XCOM**	**EXP.**	**XCOM**	**EXP.**
121.782	0.250	0.283	1.463	0.320	2.274	0.429	2.879	0.486	1.678	0.370
344.279	0.176	0.194	0.178	0.112	0.240	0.167	0.276	0.180	0.198	0.137
778.904	0.125	0.141	0.075	0.085	0.087	0.097	0.090	0.117	0.080	0.064
964.079	0.113	0.111	0.067	0.059	0.075	0.067	0.074	0.098	0.072	0.063
1085.869	0.106	0.086	0.059	0.061	0.067	0.057	0.066	0.070	0.063	0.062
1112.074	0.105	0.068	0.058	0.062	0.066	0.058	0.065	0.069	0.062	0.060
1408.006	0.093	0.062	0.050	0.041	0.056	0.054	0.055	0.055	0.053	0.055
**Sample ID**	**PS-PEG (10,000)-S0**		**PS-PEG (10,000)-S1**		**PS-PEG (10,000)-S2**		**PS-PEG (10,000)-S3**		**PS-PEG (10,000)-S4**	
	$\mu_m\ (cm^2/g)$									
**Photon Energy (keV)**	**XCOM**	**EXP.**	**XCOM**	**EXP.**	**XCOM**	**EXP.**	**XCOM**	**EXP.**	**XCOM**	**EXP.**
121.782	0.183	0.177	2.953	0.609	2.474	0.175	2.714	0.348	2.071	0.361
344.279	0.129	0.156	0.360	0.282	0.261	0.155	0.261	0.189	0.244	0.202
778.904	0.091	0.071	0.150	0.156	0.095	0.113	0.085	0.082	0.099	0.086
964.079	0.081	0.077	0.135	0.081	0.082	0.061	0.070	0.047	0.088	0.056
1085.869	0.078	0.062	0.118	0.107	0.073	0.072	0.063	0.051	0.078	0.061
1112.074	0.077	0.057	0.117	0.078	0.071	0.076	0.061	0.041	0.076	0.078
1408.006	0.682	0.044	0.101	0.053	0.061	0.040	0.052	0.041	0.066	0.054

Table S4. The mass attenuation coefficients (MACs) of the PbO doped the crosslinked PS-b-PEG-BN block copolymers samples.

**Sample ID**	**PS-PEG (1000)-BN-S0**		**PS-PEG (1000)-BN-S1**		**PS-PEG (1000)-BN-S2**		**PS-PEG (1000)-BN-S3**	
	$\mu_m\ (cm^2/g)$							
**Photon Energy (keV)**	**XCOM**	**EXP.**	**XCOM**	**EXP.**	**XCOM**	**EXP.**	**XCOM**	**EXP.**
121.782	0.173	0.420	1.814	0.268	2.456	0.437	2.185	0.470
344.279	0.122	0.173	0.190	0.095	0.235	0.167	0.242	0.146

778.904	0.086	0.126	0.069	0.052	0.076	0.093	0.093	0.112
964.079	0.078	0.079	0.057	0.051	0.062	0.075	0.079	0.097
1085.869	0.073	0.060	0.052	0.062	0.056	0.070	0.072	0.083
1112.074	0.073	0.067	0.051	0.045	0.055	0.059	0.071	0.078
1408.006	0.064	0.059	0.044	0.047	0.047	0.036	0.061	0.042
**Sample ID**	**PS-PEG (1500)-BN-S0**		**PS-PEG (1500)-BN-S1**		**PS-PEG (1500)-BN-S2**		**PS-PEG (1500)-BN-S3**	
**Photon Energy (keV)**	μ_m (cm^2/g)							
	**XCOM**	**EXP.**	**XCOM**	**EXP.**	**XCOM**	**EXP.**	**XCOM**	**EXP.**
121.782	0.145	0.151	2.044	0.468	2.050	0.389	2.470	0.529
344.279	0.103	0.128	0.214	0.206	0.196	0.150	0.274	0.245
778.904	0.073	0.078	0.077	0.096	0.064	0.069	0.105	0.143
964.079	0.066	0.069	0.065	0.074	0.052	0.050	0.089	0.099
1085.869	0.062	0.068	0.059	0.067	0.047	0.052	0.081	0.111
1112.074	0.061	0.066	0.058	0.077	0.046	0.043	0.080	0.116
1408.006	0.054	0.052	0.050	0.056	0.039	0.033	0.069	0.042
**Sample ID**	**PS-PEG (10,000)-BN-S0**		**PS-PEG (10,000)-BN-S1**		**PS-PEG (10,000)-BN-S2**		**PS-PEG (10,000)-BN-S3**	
**Photon Energy (keV)**	μ_m (cm^2/g)							
	**XCOM**	**EXP.**	**XCOM**	**EXP.**	**XCOM**	**EXP.**	**XCOM**	**EXP.**
121.782	0.156	0.142	2.367	0.312	2.456	0.286	1.556	0.374
344.279	0.108	0.094	0.248	0.224	0.235	0.171	0.173	0.151
778.904	0.076	0.080	0.090	0.109	0.076	0.075	0.066	0.067
964.079	0.069	0.061	0.075	0.055	0.062	0.068	0.056	0.051
1085.869	0.065	0.067	0.068	0.055	0.056	0.065	0.051	0.060
1112.074	0.064	0.061	0.067	0.091	0.055	0.072	0.050	0.042
1408.006	0.057	0.052	0.057	0.041	0.047	0.031	0.043	0.031

S4.2. Half-Value Layer (HVL), Tenth Value Layer (TVL), Mean Free Path (MFP), and Radiation Protection Efficiency (RPE)

Table S5. The half value layer (HVL) and the tenth value layer (TVL) values of the PbO doped the crosslinked PS-b-PEG block copolymers samples.

**Sample ID**	**PS-PEG (1000)-S0**		**PS-PEG (1000)-S1**		**PS-PEG (1000)-S2**		**PS-PEG (1000)-S3**		**PS-PEG (1000)-S4**	
**Photon Energy (keV)**	**HVL(cm)**	**TVL(cm)**	**HVL(cm)**	**TVL(cm)**	**HVL(cm)**	**TVL(cm)**	**HVL(cm)**	**TVL(cm)**	**HVL(cm)**	**TVL(cm)**
121.782	2.881	9.569	1.746	5.799	0.842	2.798	0.490	1.629	0.984	3.268
344.279	3.421	11.364	3.144	10.445	2.133	7.084	1.464	4.863	3.541	11.762
778.904	4.930	16.376	4.433	14.725	4.059	13.482	3.596	11.945	4.090	13.585
964.079	6.926	23.009	8.945	29.715	5.070	16.841	4.072	13.527	6.585	21.874
1085.869	7.381	24.518	8.332	27.679	6.092	20.236	4.169	13.848	6.259	20.791
1112.074	8.432	28.010	8.169	27.137	6.524	21.671	5.306	17.625	6.669	22.155
1408.006	8.458	28.097	9.249	30.725	5.963	19.809	6.282	20.869	6.407	21.282
**Sample ID**	**PS-PEG (1500)-S0**		**PS-PEG (1500)-S1**		**PS-PEG (1500)-S2**		**PS-PEG (1500)-S3**		**PS-PEG (1500)-S4**	
**Photon Energy (keV)**	**HVL(cm)**	**TVL(cm)**	**HVL(cm)**	**TVL(cm)**	**HVL(cm)**	**TVL(cm)**	**HVL(cm)**	**TVL(cm)**	**HVL(cm)**	**TVL(cm)**

121.782	2.457	8.162	1.407	4.673	1.031	3.426	0.667	2.217	1.176	3.905
344.279	3.592	11.934	4.040	13.421	2.644	8.784	1.807	6.004	3.174	10.545
778.904	4.929	16.375	5.286	17.558	4.546	15.103	2.779	9.233	6.758	22.450
964.079	6.243	20.739	7.635	25.364	6.567	21.816	3.298	10.955	6.900	22.920
1085.869	8.125	26.990	7.421	24.653	7.753	25.756	4.606	15.300	7.023	23.330
1112.074	10.209	33.913	7.326	24.336	7.637	25.371	4.659	15.476	7.221	23.986
1408.006	11.266	37.424	11.007	36.564	8.180	27.174	5.856	19.451	7.963	26.452
**Sample ID**	**PS-PEG (10,000)-S0**		**PS-PEG (10,000)-S1**		**PS-PEG (10,000)-S2**		**PS-PEG (10,000)-S3**		**PS-PEG (10,000)-S4**	
**Photon Energy (keV)**	**HVL(cm)**	**TVL(cm)**	**HVL(cm)**	**TVL(cm)**	**HVL(cm)**	**TVL(cm)**	**HVL(cm)**	**TVL(cm)**	**HVL(cm)**	**TVL(cm)**
121.782	3.563	11.837	0.812	2.698	2.267	7.529	0.971	3.225	1.138	3.780
344.279	4.032	13.393	1.752	5.820	2.554	8.483	1.789	5.943	2.038	6.771
778.904	8.936	29.683	3.177	10.554	3.518	11.685	4.111	13.656	4.781	15.881
964.079	8.146	27.061	6.102	20.271	6.482	21.533	7.143	23.729	7.297	24.241
1085.869	10.177	33.808	4.626	15.368	5.479	18.199	6.641	22.061	6.746	22.410
1112.074	11.104	36.886	6.330	21.028	5.200	17.272	8.185	27.189	5.234	17.386
1408.006	14.223	47.249	9.324	30.974	9.857	32.745	8.203	27.250	7.629	25.343

Table S6. The half value layer (HVL) and the tenth value layer (TVL) values of the PbO doped the crosslinked PS-b-PEG-BN block copolymers samples.

**Sample ID**	**PS-PEG (1000)-BN-S0**		**PS-PEG (1000)-BN-S1**		**PS-PEG (1000)-BN-S2**		**PS-PEG (1000)-BN-S3**	
**Photon Energy (keV)**	**HVL(cm)**	**TVL(cm)**	**HVL(cm)**	**TVL(cm)**	**HVL(cm)**	**TVL(cm)**	**HVL(cm)**	**TVL(cm)**
121.782	1.336	4.439	1.252	4.159	0.753	2.502	0.728	2.417
344.279	3.238	10.757	3.541	11.762	1.975	6.561	2.344	7.786
778.904	4.456	14.803	6.486	21.545	3.558	11.819	3.061	10.168
964.079	7.137	23.709	6.489	21.557	4.407	14.640	3.525	11.711
1085.869	9.305	30.909	5.413	17.981	4.712	15.652	4.146	13.772
1112.074	8.414	27.951	7.540	25.049	5.599	18.600	4.376	14.535
1408.006	9.462	31.431	7.128	23.677	9.260	30.760	8.087	26.863
**Sample ID**	**PS-PEG (1500)-BN-S0**		**PS-PEG (1500)-BN-S1**		**PS-PEG (1500)-BN-S2**		**PS-PEG (1500)-BN-S3**	
**Photon Energy (keV)**	**HVL(cm)**	**TVL(cm)**	**HVL(cm)**	**TVL(cm)**	**HVL(cm)**	**TVL(cm)**	**HVL(cm)**	**TVL(cm)**
121.782	2.662	8.844	0.694	2.306	0.820	2.722	0.865	2.875
344.279	3.150	10.462	1.580	5.248	2.130	7.075	1.868	6.205
778.904	5.171	17.177	3.384	11.243	4.622	15.353	3.199	10.628
964.079	5.849	19.429	4.393	14.595	6.332	21.034	4.606	15.301
1085.869	5.872	19.505	4.845	16.096	6.170	20.496	4.108	13.647
1112.074	6.070	20.165	4.237	14.076	7.332	24.356	3.953	13.132
1408.006	7.801	25.913	5.767	19.159	9.648	32.050	11.010	36.574
**Sample ID**	**PS-PEG (10,000)-BN-S0**		**PS-PEG (10,000)-BN-S1**		**PS-PEG (10,000)-BN-S2**		**PS-PEG (10,000)-BN-S3**	
**Photon Energy (keV)**	**HVL(cm)**	**TVL(cm)**	**HVL(cm)**	**TVL(cm)**	**HVL(cm)**	**TVL(cm)**	**HVL(cm)**	**TVL(cm)**

121.782	3.721	12.359	0.967	3.213	1.105	3.672	1.070	3.553
344.279	5.616	18.656	1.345	4.469	1.848	6.139	2.647	8.794
778.904	6.649	22.086	2.781	9.237	4.213	13.994	5.984	19.878
964.079	8.689	28.863	5.480	18.203	4.630	15.378	7.854	26.090
1085.869	7.957	26.433	5.472	18.176	4.868	16.172	6.702	22.264
1112.074	8.702	28.906	3.322	11.036	4.375	14.533	9.521	31.628
1408.006	10.112	33.592	7.347	24.405	10.350	34.381	12.765	42.404

Table S7. The mean free path (MFP) and the radiation protection efficiency (RPE) values of the PbO doped the crosslinked PS-b-PEG block copolymers samples.

**Sample ID**	**PS-PEG (1000)-S0**		**PS-PEG (1000)-S1**		**PS-PEG (1000)-S2**		**PS-PEG (1000)-S3**		**PS-PEG (1000)-S4**	
**Photon Energy (keV)**	**MFP(cm)**	**RPE(%)**	**MFP(cm)**	**RPE(%)**	**MFP(cm)**	**RPE(%)**	**MFP(cm)**	**RPE(%)**	**MFP(cm)**	**RPE(%)**
121.782	4.156	10.219	2.518	16.397	1.215	28.521	0.707	32.497	1.420	33.866
344.279	4.935	8.678	4.536	9.464	3.077	12.420	2.112	12.333	5.108	10.856
778.904	7.112	6.105	6.395	6.809	5.855	6.731	5.188	5.218	5.900	9.470
964.079	9.993	4.384	12.905	3.434	7.314	5.426	5.875	4.622	9.500	5.992
1085.869	10.648	4.120	12.021	3.682	8.788	4.537	6.014	4.517	9.029	6.294
1112.074	12.165	3.616	11.785	3.755	9.411	4.243	7.654	3.567	9.622	5.918
1408.006	12.203	3.605	13.344	3.323	8.603	4.632	9.063	3.021	9.243	6.154
**Sample ID**	**PS-PEG (1500)-S0**		**PS-PEG (1500)-S1**		**PS-PEG (1500)-S2**		**PS-PEG (1500)-S3**		**PS-PEG (1500)-S4**	
**Photon Energy (keV)**	**MFP(cm)**	**RPE(%)**	**MFP(cm)**	**RPE(%)**	**MFP(cm)**	**RPE(%)**	**MFP(cm)**	**RPE(%)**	**MFP(cm)**	**RPE(%)**
121.782	3.545	12.960	2.029	24.562	1.488	28.157	0.963	31.265	1.696	31.596
344.279	5.183	9.057	5.829	9.348	3.815	12.100	2.607	12.930	4.579	13.119
778.904	7.111	6.685	7.626	7.227	6.559	7.227	4.010	8.610	9.750	6.392
964.079	9.007	5.316	11.015	5.060	9.475	5.060	4.758	7.307	9.954	6.265
1085.869	11.722	4.111	10.707	5.202	11.186	4.303	6.645	5.288	10.132	6.158
1112.074	14.728	3.285	10.569	5.268	11.019	4.367	6.721	5.229	10.417	5.995
1408.006	16.253	2.982	15.879	3.538	11.802	4.083	8.448	4.183	11.488	5.452
**Sample ID**	**PS-PEG (10,000)-S0**		**PS-PEG (10,000)-S1**		**PS-PEG (10,000)-S2**		**PS-PEG (10,000)-S3**		**PS-PEG (10,000)-S4**	
**Photon Energy (keV)**	**MFP(cm)**	**RPE(%)**	**MFP(cm)**	**RPE(%)**	**MFP(cm)**	**RPE(%)**	**MFP(cm)**	**RPE(%)**	**MFP(cm)**	**RPE(%)**
121.782	5.141	11.241	1.172	23.439	3.270	11.731	1.401	24.841	1.642	26.167
344.279	5.816	10.003	2.527	11.648	3.684	10.483	2.581	14.356	2.941	15.580
778.904	12.891	4.644	4.584	6.601	5.075	7.725	5.931	6.522	6.897	6.966
964.079	11.752	5.082	8.804	3.493	9.352	4.269	10.306	3.807	10.528	4.620
1085.869	14.683	4.089	6.674	4.581	7.904	5.031	9.581	4.089	9.732	4.988
1112.074	16.019	3.754	9.132	3.369	7.501	5.294	11.808	3.331	7.551	6.383
1408.006	20.520	2.943	13.452	2.300	14.221	2.828	11.835	3.323	11.006	4.424

Table S8. The mean free path (MFP) and the radiation protection efficiency (RPE) values of the PbO doped the crosslinked PS-b-PEG-BN block copolymers samples.

**Sample ID**	**PS-PEG (1000)-BN-S0**		**PS-PEG (1000)-BN-S1**		**PS-PEG (1000)-BN-S2**		**PS-PEG (1000)-BN-S3**	
**Photon Energy (keV)**	**MFP(cm)**	**RPE(%)**	**MFP(cm)**	**RPE(%)**	**MFP(cm)**	**RPE(%)**	**MFP(cm)**	**RPE(%)**

121.782	1.928	23.840	1.806	22.438	1.087	31.930	1.050	30.375
344.279	4.672	10.629	5.108	8.594	2.850	13.644	3.381	10.629
778.904	6.429	7.842	9.357	4.787	5.133	7.821	4.416	8.245
964.079	10.297	4.971	9.362	4.785	6.358	6.363	5.086	7.199
1085.869	13.424	3.836	7.809	5.708	6.798	5.964	5.981	6.156
1112.074	12.139	4.233	10.879	4.132	8.078	5.043	6.313	5.843
1408.006	13.651	3.773	10.283	4.366	13.359	3.081	11.667	3.205
**Sample ID**	**PS-PEG (1500)-BN-S0**		**PS-PEG (1500)-BN-S1**		**PS-PEG (1500)-BN-S2**		**PS-PEG (1500)-BN-S3**	
**Photon Energy (keV)**	**MFP(cm)**	**RPE(%)**	**MFP(cm)**	**RPE(%)**	**MFP(cm)**	**RPE(%)**	**MFP(cm)**	**RPE(%)**
121.782	3.841	10.894	1.002	33.126	1.182	34.154	1.249	30.206
344.279	4.544	9.290	2.279	16.206	3.073	14.851	2.695	15.347
778.904	7.460	5.766	4.883	7.922	6.668	7.141	4.616	9.270
964.079	8.438	5.115	6.338	6.160	9.135	5.264	6.645	6.534
1085.869	8.471	5.095	6.990	5.602	8.901	5.399	5.927	7.296
1112.074	8.758	4.933	6.113	6.380	10.578	4.563	5.703	7.571
1408.006	11.254	3.860	8.321	4.728	13.919	3.487	15.884	2.787
**Sample ID**	**PS-PEG (10,000)-BN-S0**		**PS-PEG (10,000)-BN-S1**		**PS-PEG (10,000)-BN-S2**		**PS-PEG (10,000)-BN-S3**	
**Photon Energy (keV)**	**MFP(cm)**	**RPE(%)**	**MFP(cm)**	**RPE(%)**	**MFP(cm)**	**RPE(%)**	**MFP(cm)**	**RPE(%)**
121.782	5.368	9.890	1.395	20.438	1.595	22.914	1.543	33.647
344.279	8.102	6.667	1.941	15.157	2.666	14.415	3.819	15.274
778.904	9.592	5.661	4.012	7.644	6.078	6.601	8.633	7.070
964.079	12.535	4.362	7.905	3.955	6.679	6.025	11.331	5.433
1085.869	11.480	4.753	7.894	3.961	7.024	5.738	9.669	6.337
1112.074	12.554	4.355	4.793	6.439	6.312	6.364	13.736	4.504
1408.006	14.589	3.759	10.599	2.965	14.931	2.741	18.416	3.379

Table S9. Comparative informations of PbO doped radiation shielding characteristics.

Authors	Sample Information	Main Consequences
This research	PS-PEG (1000-1500-10,000)-S0,1,2,3,4<br>PS-PEG (1000-1500-10,000)-BN-S0,1,2,3	It was observed that the implemented radiation does not influence the structure of the materials and the radiation protection capacities of the samples improved when the PbO or BN, and PS-b-PEG copolymer percentages of the prepared materials were changed.
Cinan, Z.M.; et al. [56]	Cement-HB(PAE-b-PCL)-PU Plaster-Lead Oxide PCMs<br>Cement-HB(PAE-b-PCL)-PU Plaster-Arsenic Oxide PCMs	The values of HVL, TVL, and MFP decrease with an increase in weight fraction of high atomic number elements in the material due to an increase in MACs and intensity. It was observed by Cinan et al. that deduced that when the proportion of the

		lead and arsenic oxide ingredient in the PCMs is ascent, the absorption proportion and hence the safety qualities of the PCM sample ascents. Their results, the utility of lead oxide doped materials in gamma-ray shielding is greater than that of arsenic oxide doped materials.
Hassan et al. [28]	Concrete Incorporation with Two Types of Nano-Lead Compounds	Hassan et al. investigated that the influence of concrete mixing with two kinds of nano-lead composites on its gamma absorbing qualities and they have comprehensively expressed how the gamma attenuation coefficients of nano-lead oxide added concrete change at different energies, the investigation results indicated that the addition of nano-lead oxide to concrete can ascent its mass attenuation coefficients to reach 33% of pure lead plates at 662 keV.
Kozlovskiy et al. [29]	$0.5Bi_2O_3$-(0.5-x)WO_3-xPbO Glasses	PbO is one of the appropriate metal oxides for protection from high-energy gamma rays' harmful effects. It was noticed by Kozlovskiy et al. that the substitution of Pb atoms for W atoms leads to a lessen in the half value layer values by 44%, 65%, and 73% for various lead oxide concentrations and found that an increase in the lead oxide concentration leads to a remarkable decrease in the required density for attenuation by 50% of the density of gamma irradiation.
Mahmoud et al. [30]	xBaO-(50-x)PbO–$50P_2O_5$ (x = 0, 5, 10, 15, 20, 30, 40, 50 mol%)	Mahmoud et al. observed that the decline in the lead oxide percentage has an unfavorable influence on the mass attenuation coefficients.
Mokhtari, K.; et al. [31]	The Ordinary Concrete Reinforced with Different Weight Fractions of PbO Micro/Nanoparticles	The experimental results illustrated by Mokhtari et al. that the half value later coefficient of the standard concrete strengthened with 5 wt% of nano lead oxide particles was decreased by 64% at 511 keV and 48% at 1332 keV and good harmony was observed among simulation and experimental results and indicated that

		the employment of nano lead oxide is more powerful at low gamma energies up to 1MeV.
Othman, S.A.; [32]	Lead Oxide on Concrete	It was observed by Othman that all results for various samples demonstrated that the intensity of gamma-ray was reduced with the ascent in the density of concrete and the stiffness of concrete ascent as the composition of lead rising.
Qian, Z.; et al. [33]	Sm^{3+} Doped (60-x)B_2O_3-xPbO-14TeO_2-10P_2O_5-7.5BaO-7.5CdO-1Sm_2O_3 (x=0, 10, 20, 30 and 40 wt%) Glass Systems	Qian et al. investigated the effect of PbO content on the gamma-ray attenuation coefficients of Sm^{3+} doped (60-x)B_2O_3-xPbO-14TeO_2-10P_2O_5-7.5BaO-7.5CdO-1Sm_2O_3 (x=0, 10, 20, 30, and 40 wt%) glass system among 0.02–10 MeV photon energy range and simulation results are fine harmony with the theoretical XCOM calculations in this work, the outcomes demonstrate that the increasing percentage of lead oxide in the glass combinations can develop the gamma-ray shielding capacity of the investigated glasses.
Alotaibi, B.M.; et al. [34]	40P_2O_5-20CaO-(30-x)Na_2O-10K_2O-xPbO Glass Systems (x = 0, 5, 10, 15, 20 mol%)	Alotaibi et al. prepared the glasses of composition of 40P_2O_5-20CaO-(30-x)Na_2O-10K_2O-xPbO (for x = 0, 5, 10, 15, and 20 mol%), observed the physical properties and the optical properties, and found to be influenced by the combination of the PbO, their results indicate that PbP5, the glass with the biggest lead oxide content, has the greatest linear attenuation coefficient at all examined energy regions, and thus the best shielding potentiality.
Alzahrani et al. [35]	PbO–B_2O_3–ZnO Glass Systems (40PbO–50B_2O_3–10ZnO, 30PbO–50B_2O_3–10Bi_2O_3–10ZnO, 20PbO–50B_2O_3–20Bi_2O_3–10ZnO)	Alzahrani et al. investigated that the influence of altering PbO with Bi_2O_3 on the physical, mechanical, and radiation shielding characteristics of a PbO–B_2O_3–ZnO glass system and found that the results of the radiation shielding calculations for the changing of PbO with Bi_2O_3 a little bit reduced the radiation shielding competence.

El-Mallawany et al. [36]	{[$(TeO_2)_{0.7}$-$(PbO)_{0.3}$]$_{1-x}$$(ZnO)_x$} (x=0.15–0.25 mol%) Glass Combinations	El-Mallawany et al. studied the influence of PbO and TeO_2 composition on the photon, fast neutron, and charged particles shielding ability of the triple ZnO-PbO-TeO_2 glass combinations with the form [$(TeO_2)_{0.7}$-$(PbO)_{0.3}$]$_{1-x}$$(ZnO)_x$ (for x=0.15–0.25 mol%) glass structure and they found that these samples can also be a beneficial shielding material for fast neutrons and alpha-proton particles and will be beneficial in the probable application of Te-based glasses for shielding applications.
Al-Harby et al. [37]	{$40P_2O_5$–20CaO–(30−x)Na_2O−$10K_2O$–xPbO} (x = 0, 5, 10, 15, 20 mol%) Glass Systems	Al-Harby et al.'s study is about the synthesis, physical, structural, and gamma-ray shielding characteristics of $40P_2O_5$–20CaO–(30−x)Na_2O−$10K_2O$–xPbO (for x = 0, 5, 10, 15, 20 mol%) glasses and they found that the sample' shielding strength improved as PbO content ascended and the calculated radiation shielding coefficients of the samples illustrated that they possess a decent potential for radiation shielding implementations.
Al-Buriahi et al. [38]	$50GeO_2$-(50-x)PbO-xZnO (x = 0, 10, 20, 30, 40, 50 mol%)	Al-Buriahi et al. researched that on the effects of reducing PbO content on the elastic and radiation shielding properties of germanate glasses described by the chemical formula $50GeO_2$-(50-x)PbO-xZnO (where x values change between 0 and 50 mol % with the step of 10) and they found that these glass samples have high photon absorbing skill compared to standard and barite concrete, their results illustrated that the GeO_2-PbO-ZnO glass structure can be used as a non-toxic shielding matter in nuclear facilities.
Almuqrin, A.H.; et al. [57]	PbO-WO_3-Na_2O-MgO-B_2O_3 Glasses	Almuqrin et al.'s results demonstrated that the rising of PbO ingredients of substances had an affirmative influence on shielding substances' efficiency.

Table S10. Comparative informations of boron-doped radiation shielding characteristics.

Authors	Sample Information	Main Consequences
This research	PS-PEG (1000-1500-10,000)-S0,1,2,3,4<br>PS-PEG (1000-1500-10,000)-BN-S0,1,2,3	It was observed that the implemented radiation does not influence the structure of the materials and the radiation protection capacities of the samples improved when the PbO or BN, and PS-b-PEG copolymer percentages of the prepared materials were changed.
Stalin et al. [39]	$20Li_2O$-(20-x)Bi_2O_3-xWO_3-60B_2O_3<br>(x=0, 1, 2, 3, 4, 5 mol%)	Stalin et al. researched the optical, mechanical, chemical durability, and gamma irradiation shielding characteristics of six different lithium bismuth boro-tungstate glass samples with chemical structure $20Li_2O$-(20-x)Bi_2O_3-xWO_3-60B_2O_3 (x=0, 1, 2, 3, 4, 5 mol%) and they found that LBWB glass materials could be used in high energy nuclear radiation exposure are such as nuclear research institution, nuclear energy production centers and many other places for radiation shielding implementations.
Algradee et al. [40]	{(100-x)[20Li_2O-10ZnO-20B_2O_3-50P_2O_5]-xNd_2O_3}<br>(x=0.0 [LZBP:0.0Nd] wt%)<br>(x=4.0 [LZBP:4.0Nd] wt%)	Algradee et al. investigated that Lithium-zinc-borophosphate glasses with the composition of (100-x)[20Li_2O-10ZnO-20B_2O_3-50P_2O_5]-xNd_2O_3 (here x = 0.0 (LZBP:0.0Nd) to 4.0 (LZBP:4.0Nd) wt%) and found that the glass LZBP:4.0Nd has the lowest half value layer and hence the high shielding ability for gamma radiations.
Jiao et al. [41]	Hexagonal Boron Nitride (*h*-BN)<br>*h*-BN/EP Composites	Jiao et al. said that to improve the radiation resistance of epoxy resin is important to illustrate the reliability of equipment and the safety of facilities in the nuclear industry, their results showed that the blending with *h*-BN ascent the radiation resistance characteristics of epoxy resin and the neutron shielding capability.
Fu et al. [42]	Polymer-Based Composites, High-Density Concrete, Heavy Metals, Paraffin, and Other Neutron Shielding Materials with Additives (*h*-BN, B_4C,	Fu et al. reviewed the newest neutron shielding substances for the storage of nuclear fuel waste including contributions such as boron carbide (B_4C), boron nitride

	Boric Acid, Colemanite, Cd, Gd, Sm_2O_3) Fillers	(BN), boric acid (H_3BO_3), and colemanite. They provided a theoretical groundwork for the optimization scheme and experimental arrangement of neutron shielding materials.
